# Lurasidone compared to other atypical antipsychotic monotherapies for adolescent schizophrenia: a systematic literature review and network meta-analysis

**DOI:** 10.1007/s00787-019-01425-2

**Published:** 2019-11-22

**Authors:** Celso Arango, Daisy Ng-Mak, Elaine Finn, Aidan Byrne, Antony Loebel

**Affiliations:** 1 Department of Child and Adolescent Psychiatry, Institute of Psychiatry and Mental Health, Hospital General Universitario Gregorio Marañón. IiSGM, School of Medicine, Universidad Complutense, CIBERSAM. Av. Séneca 2, 28040 Madrid, Spain; 2 Sunovion Pharmaceuticals Inc, 84 Waterford Drive, Marlborough, MA 01752 USA; 3 IQVIA, 210 Pentonville Rd, London, N1 9JY UK; 4 Sunovion Pharmaceuticals Inc, One Bridge Plaza North, Suite 510, Fort Lee, NJ 07024 USA

**Keywords:** Schizophrenia, Adolescent, Network meta-analysis, Lurasidone, Body weight changes

## Abstract

This network meta-analysis assessed the efficacy and tolerability of lurasidone versus other oral atypical antipsychotic monotherapies in adolescent schizophrenia. A systematic literature review identified 13 randomized controlled trials of antipsychotics in adolescents with schizophrenia-spectrum disorders. A Bayesian network meta-analysis compared lurasidone to aripiprazole, asenapine, clozapine, olanzapine, paliperidone extended-release (ER), quetiapine, risperidone, and ziprasidone. Outcomes included Positive and Negative Syndrome Scale (PANSS), Clinical Global Impressions-Severity (CGI-S), weight gain, all-cause discontinuation, extrapyramidal symptoms (EPS), and akathisia. Results were reported as median differences for continuous outcomes and odds ratios (ORs) for binary outcomes, along with 95% credible intervals (95% CrI). Lurasidone was significantly more efficacious than placebo on the PANSS (− 7.95, 95% CrI − 11.76 to − 4.16) and CGI-S (− 0.44, 95% CrI − 0.67 to − 0.22) scores. Lurasidone was associated with similar weight gain to placebo and statistically significantly less weight gain versus olanzapine (− 3.62 kg, 95% CrI − 4.84 kg to − 2.41 kg), quetiapine (− 2.13 kg, 95% CrI − 3.20 kg to − 1.08 kg), risperidone (− 1.16 kg, 95% CrI − 2.14 kg to − 0.17 kg), asenapine (− 0.98 kg, 95% CrI − 1.71 kg to − 0.24 kg), and paliperidone ER (− 0.85 kg, 95% CrI − 1.57 kg to − 0.14 kg). The odds of all-cause discontinuation were significantly lower for lurasidone than aripiprazole (OR = 0.28, 95% CrI 0.10–0.76) and paliperidone ER (OR = 0.25, 95% CrI 0.08–0.81) and comparable to other antipsychotics. Rates of EPS and akathisia were similar for lurasidone and other atypical antipsychotics. In this network meta-analysis of atypical antipsychotics in adolescent schizophrenia, lurasidone was associated with similar efficacy, less weight gain, and lower risk of all-cause discontinuation compared to other oral atypical antipsychotics.

**Electronic supplementary material:**

The online version of this article (10.1007/s00787-019-01425-2) contains supplementary material, which is available to authorized users.

## Introduction

Schizophrenia is a severe, chronic, disabling mental illness associated with pronounced psychiatric symptoms, physical comorbidities, and increased mortality rates [1, 2]. In adult patients with schizophrenia, maintaining activities of daily life can be challenging, and few patients are able to maintain employment, live independently, or maintain a marital/partner relationship [3]. Schizophrenia can manifest prior to adulthood with an estimated prevalence of 0.5% among adolescents aged 13–17 years [2], while childhood onset schizophrenia (age ≤ 12) is very rare [4]. Early onset patients tend to have more severe negative symptoms, cognitive impairment, impulsivity [5], frequent hospitalizations [6], and poor social functioning [7]. Several lines of evidence suggest that patients with schizophrenia may accrue progressive chronicity and disability over time such that, at least theoretically, promptly mitigating symptoms could potentially impede disease progression [8, 9].

Atypical antipsychotics are recommended over typical antipsychotics as first-line treatment for adolescent patients with schizophrenia [1] due to a reduced risk of extrapyramidal symptoms (EPS) and akathisia [1, 10]. However, the atypical antipsychotics are not a homogenous group of medications and have varied tolerability profiles that can include weight gain, lipid abnormalities, glucose abnormalities, prolactin elevation, or sedation [10]. Guidelines recommend carefully considering the tolerability profile when selecting an antipsychotic with a specific patient [10, 11]. Adolescent patients appear particularly susceptible to metabolic issues, including long-term risk of diabetes and hyperlipidemia [1]; therefore, treatment guidelines recommend ongoing weight and metabolic monitoring when using antipsychotics in this population [1, 10, 11].

Understanding how atypical antipsychotic treatment impacts the vulnerable adolescent patient population is imperative, not only because prompt, effective treatment can lead to better long-term outcomes [9], but also because adolescents may be exposed to atypical antipsychotics for many years. There are very few randomized head-to-head clinical trials comparing atypical antipsychotics’ efficacy and tolerability in patients under the age of 18 [12, 13]. Lurasidone was recently approved for schizophrenia in adolescents in the United States. No head-to-head trials have yet compared lurasidone to any other atypical antipsychotics in this population. Three prior network meta-analyses examining antipsychotics in adolescents and children with schizophrenia have been previously published [14–16]. Only the most recent one [16], which was published after this project was initiated, included lurasidone. This recent study [16] also included older trials of typical antipsychotics. Replication and consistency of findings, particularly when using varied methods and assumptions, is crucial in science [17]. The objective of this study was to estimate the relative efficacy and tolerability of lurasidone monotherapy versus other atypical antipsychotic monotherapies for the management of adolescent schizophrenia through network meta-analysis.

## Methods

### Literature search and selection

A systematic literature review using the preferred reporting items for systematic reviews and meta-analyses (PRISMA) statement [18], aligned with the Centre for Reviews and Dissemination guide for conducting systematic reviews [19], was conducted, with searches run on June 4, 2016. The searches identified randomized controlled trials of atypical antipsychotic monotherapy that included an adolescent schizophrenia population. Trials included adolescent patients (13–17 years of age) who were diagnosed with schizophrenia or other schizophrenia-spectrum disorders (i.e., schizoaffective disorder, schizophreniform disorder, and psychosis not otherwise specified). Searches were run in Embase (1988 through week 13 of 2016), Ovid MEDLINE (1946 through June 4, 2016), and Cochrane Library (1991 through first quarter of 2016). Eligibility criteria for trial inclusion were designed using the population, intervention, comparison, outcome measures, and study design (PICOS) criteria [20]. Only articles published in English were included. Abstracts were first reviewed for eligibility. Next, full-text articles were obtained and reviewed to determine their consistency with the pre-specified criteria. If there was uncertainty about including a study, a second independent reviewer was consulted.

A quality assessment was performed for all included trials using the Cochrane Collaboration’s tool for assessing risk of bias [21]. Quality assessment indicated the strength and robustness of the evidence available and evaluated potential different sources of bias such as selection bias, performance bias, measurement bias and attrition bias. Details of the systematic review and quality assessment are included in Online Appendix 1.

### Network meta-analysis

A network meta-analysis allows differences between antipsychotics that have not been directly evaluated to be examined through a common comparator. The network meta-analysis included clinical trials of acute antipsychotic treatment for schizophrenia-spectrum disorders in adolescents and was conducted with a Bayesian framework using WinBUGS version 1.4.3 [22]. The efficacy outcome measures included change from baseline in Positive and Negative Syndrome Scale (PANSS) score and change from baseline in Clinical Global Impressions-Severity Scale (CGI-S) score. The main tolerability outcome variables included change from baseline in body weight, all-cause treatment discontinuation, EPS, and akathisia. Results for other outcome variables are reported in Online Appendix 2: response rate, discontinuation due to adverse events, sedation, somnolence, and change from baseline in metabolic laboratory values, including serum glucose, total cholesterol, and triglycerides. The primary analyses contrasted the atypical antipsychotics with lurasidone; comparisons with placebo are presented in Online Appendix 3. Comparisons between antipsychotics on the main efficacy and tolerability variables are presented in Online Appendix 4. The network meta-analysis was based on the primary outcome period of each trial as reported, regardless of whether treatment ended at 6, 8, or 12 weeks. This approach has been used previously in network meta-analyses conducted by the National Institute for Health and Care Excellence as part of the Clinical Guideline 185 [23]. Trials used either mixed model for repeated measures analysis (MMRM) or the last observation carried forward to handle missing data; when available, estimates from MMRM were used in the network meta-analysis. For trials with multiple fixed-dose arms with the same antipsychotic, the results were pooled by antipsychotic.

Results for continuous outcomes were expressed as the median difference in change from baseline to the final endpoint. Results for binary outcomes were expressed as odds ratios (ORs). Ninety-five percent credible intervals (95% Crls), the Bayesian analogue to confidence intervals, were reported alongside estimates. Results were interpreted as statistically significant if the 95% CrIs did not include zero for continuous outcomes or one for binary outcomes.

Both fixed- and random-effects models were analyzed for all outcomes. Model fit was assessed using deviance information criterion (DIC), a deviance-based measure that penalizes models for complexity. Results of the fixed-effects models were selected as the primary results, as they generally fit the data better based on the DIC. Results from the random-effects models are included in Online Appendix 5.

### Assessments of heterogeneity and inconsistency

Pairwise meta-analyses were conducted to assess between-trial heterogeneity for comparisons that were directly informed by more than one trial. This heterogeneity was quantified using the *I*^2^ statistic, which ranges from 0 to 100%. A high *I*^2^ (i.e., 75% or higher) implies that a significant portion of the difference in trial results could not be explained by the model. For all networks containing closed loops (direct or indirect evidence for a particular comparison), Bucher tests were performed to detect inconsistency between these sources of evidence, hence assessing the assumption of transitivity [24]. Heterogeneity and inconsistency provide an indication of impactful differences in study populations across the trials.

### Sensitivity analyses

Three sensitivity analyses were conducted to further examine the effect of trial design on the estimated treatment effects. The first sensitivity analysis excluded the two trials with a lower mean patient age at baseline: (Mozes et al. [25] and Shaw et al. [26]). The second sensitivity analysis excluded trials in which patients were treated for longer than 6 weeks. There were three trials of 8-week duration and two trials of 12-week duration (Table 1). Sequential removal of trials of 12-week duration, followed by trials of 8- and 12-week duration assessed the impact of trial duration on the base case results. Two trials directly compared risperidone and olanzapine, but reported contradictory results on all-cause discontinuation (Jensen et al. [27] and Mozes et al. [25]). In the third sensitivity analysis, these two trials were individually removed from the relevant outcome models.

**Table 1:** Trial design and patient baseline characteristics

Trial	Treatment comparisons	*n*	Treatment duration (weeks)	Baseline characteristics					
				Female (%)	Age Mean (SD)	Age at onset Mean (SD)	Weight (kg) Mean (SD)	PANSS Mean (SD)	CGI-S Mean (SD)
Goldman et al. [37]	Placebo	112	6	36.6	15.3 (1.4)	13.1 (2.7)	64.0 (11.9)	92.8 (11.1)	4.8 (0.6)
	Lurasidone 40 mg	108		38.0	15.5 (1.3)	13.4 (2.7)	63.6 (12.4)	94.5 (11.0)	4.9 (0.6)
	Lurasidone 80 mg	106		34.0	15.3 (1.4)	12.9 (2.9)	63.8 (12.9)	94.0 (11.1)	4.8 (0.7)
Findling et al. [28]	Placebo	100	6	39.0	15.4 (1.4)	14.0 (2.6)	63.4 (15.6)	95.0 (15.5)	4.6 (0.8)
	Aripiprazole10 mg	100		55.0	15.6 (1.3)	14.2 (2.5)	63.5 (19.1)	93.7 (15.7)	4.5 (0.8)
	Aripiprazole 30 mg	102		36.3	15.4 (1.4)	14.2 (2.0)	64.5 (15.5)	94.9 (15.5)	4.6 (0.6)
Findling et al. [29]	Placebo	102	8	39.2	15.4 (1.4)	13.8 (2.2)	60.5 (6.4)	97.5 (10.3)	4.6 (0.6)
	Asenapine 2.5 mg	98		36.7	15.2 (1.5)	13.2 (2.7)	58.4 (15.1)	97.4 (10.2)	4.6 (0.6)
	Asenapine 5 mg	106		36.8	15.4 (1.5)	13.4 (2.7)	62.2 (16.1)	98.6 (13.4)	4.7 (0.6)
Shaw et al. [26]	Clozapine 12.5–900 mg	12	8	33.3	11.7 (2.3)	8.6 (2.7)	NR	NR	6.0 (1.2)
	Olanzapine 5–20 mg	13		46.2	12.8 (2.4)	9.5 (2.2)	NR	NR	5.3 (0.9)
Kryzhanovskaya et al. [35]	Placebo	35	6	31.4	16.3 (1.6)	13.4 (2.8)	68.9 (16.9)	95.5 (14.1)	4.9 (0.8)
	Olanzapine 2.5–20 mg	72		29.2	16.1 (1.3)	12.5 (3.2)	67.0 (13.3)	95.3 (14.1)	4.8 (0.7)
Mozes et al. [25]	Risperidone 0.25–4.5 mg	13	12	61.5	10.7 (1.4)	9.0 (NR)	NR	93.9 (27.1)	NR
	Olanzapine 2.5–20 mg	12		58.3	11.5 (1.6)	9.1 (NR)	NR	92.8 (26.9)	NR
Singh et al. [32]	Placebo	51	6	54.9	15.7 (1.4)	13.4 (2.4)	59.5 (16.5)	90.6 (12.1)	NR
	Paliperidone ER 1.5 mg	54		17.0	15.1 (1.5)	12.5 (2.9)	60.4 (16.1)	91.6 (12.5)	NR
	Paliperidone ER 3 mg or 6 mg	48		35.4	15.3 (1.6)	13.0 (1.9)	57.7 (14.6)	90.6 (14.0)	NR
	Paliperidone ER 6 mg or 12 mg	47		29.8	15.5 (1.6)	12.8 (3.2)	61.5 (16.1)	91.5 (13.9)	NR
Savitz et al. [33]	Aripiprazole 5–15 mg	114	8	33.3	15.4 (1.5)	12.6 (2.8)	60.4 (14.6)	92.0 (12.1)	NR
	Paliperidone ER 3–9 mg	112		34.8	15.3 (1.5)	13.2 (2.1)	59.4 (15.5)	89.6 (12.2)	NR
Findling et al. [30]	Placebo	73	6	42.5	15.3 (1.4)	13.6 (3.0)	62.5 (14.4)	96.7 (18.0)	4.7 (0.7)
	Quetiapine 400 mg	73		41.1	15.5 (1.3)	13.5 (3.5)	61.0 (19.1)	96.2 (17.7)	4.7 (0.8)
	Quetiapine 800 mg	74		40.5	15.5 (1.3)	13.7 (3.1)	61.7 (14.7)	97.0 (15.3)	4.6 (0.8)
Jensen et al. [27]	Risperidone 0.5–6 mg	10	12	20.0	15.6 (2.5)	NR	NR	NR	NR
	Olanzapine 5–20 mg	10		50.0	15.3 (1.5)	NR	NR	NR	NR
	Quetiapine 100–800 mg	10		30.0	14.8 (2.3)	NR	NR	NR	NR
Haas et al. [34]	Placebo	54	6	35.2	15.5 (1.4)	14.8 (1.6)	NR	93.2 (10.3)	4.6 (0.7)
	Risperidone 1–3 mg	55		45.5	15.7 (1.3)	14.5 (2.6)	NR	95.4 (11.0)	4.7 (0.8)
	Risperidone 4–6 mg	51		27.5	15.7 (1.3)	14.8 (2.3)	NR	93.0 (11.9)	4.5 (0.7)
Findling et al. [31]	Placebo	90	6	31.1	15.4 (2.0)	NR	64.3 (15.7)	88.7 (18.7)	4.6 (0.7)
	Ziprasidone 80–160 mg	193		43.5	15.2 (1.9)	NR	61.2 (15.5)	88.1 (17.6)	4.7 (0.7)

*CGI-S* Clinical Global Impressions-Severity Scale, *NR* not reported, *PANSS* Positive and Negative Syndrome Scale, *SD* standard deviation

## Results

### Included trials

**Fig. 1:**
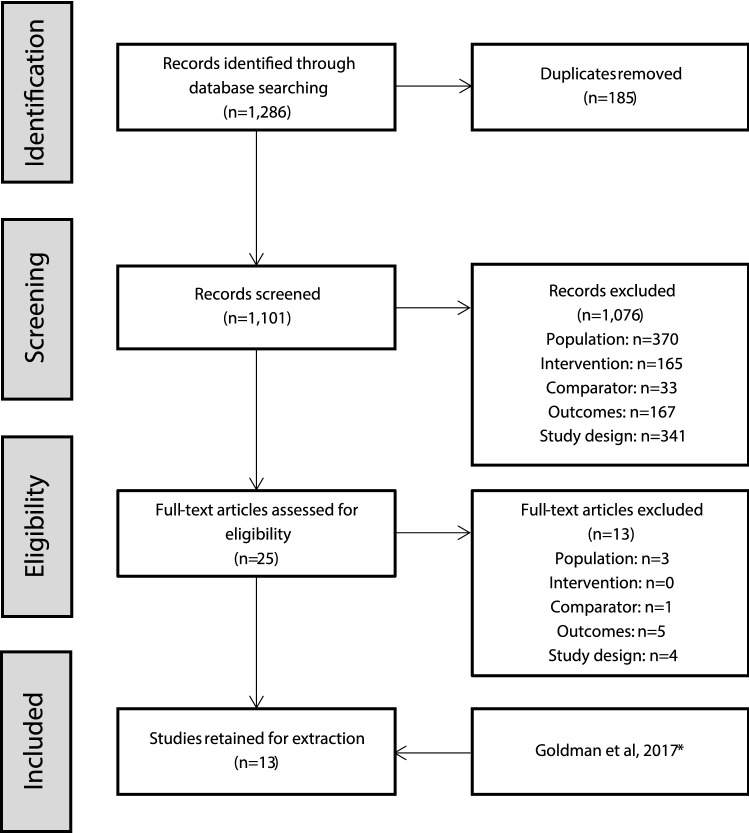
PRISMA flow diagram. Study D1050301 was not published when the literature search was conducted. It is referred to in the text either by the study identifier or the publication, Goldman et al. [37], where appropriate. One trial [36] met all of the selection criteria, but compared two dosing regimens of risperidone. When doses were pooled, it effectively became a one-arm trial and could not be linked into the network

The systematic literature search identified a total of 1286 citations, 185 of which were duplicates. After removing duplicates, the titles and abstracts of 1101 citations were screened, 25 of which were retained for full-text review (see Online Appendix 1). Results of the full-text review yielded 12 trials that met eligibility criteria [25–36]. In addition, a clinical trial report from Study D1050301 was provided by Sunovion Pharmaceuticals Inc (subsequently described by Goldman et al. [37]). One trial compared two different dosing regimens of risperidone [36] and when doses were pooled, it effectively becomes a single-armed trial and could not be linked into the network. Twelve trials were included in the meta-analysis (Fig. 1). Most participants were aged 12–17, but three trials also included patients outside of this range (Jensen et al. [27], Mozes et al. [25], and Shaw et al. [26]). Jensen et al. [27], included patients aged 10–18 years, but the population had a mean age similar to the other studies; whereas Mozes et al. [25] and Shaw et al. [26] had younger populations with mean ages ranging from 10.7 to 12.8 years. The baseline PANSS total score and CGI-S scores appeared similar across all of the included studies (Table 1). Further details of the study designs and baseline characteristics from the included trials are presented in Table 1.

### Assessments of heterogeneity and inconsistency

On comparison, the rate of all-cause discontinuation between risperidone and olanzapine, was informed by direct evidence from two studies (Mozes et al. [25]; Jensen et al. [27]). In a consistency check, these two trials presented descriptively conflicting results; the impact of which was assessed in a sensitivity analysis (Online Appendix 6). The only comparison informed by both direct and indirect evidence that demonstrated inconsistency on the Bucher tests was weight change between aripiprazole and placebo. Direct evidence from Findling et al. [28] indicated that aripiprazole led to greater weight gain relative to placebo; however, the indirect evidence (involving data from Singh et al. [32], Savitz et al. [33], and Findling et al. [28]) indicated less weight gain compared to placebo. This inconsistency suggested potential issues with the comparability of the studies comparing these treatments. This was further investigated using pairwise meta-analysis, which identified a moderately high degree of between-trial heterogeneity (*I*^2^ = 73.9%).

### Network meta-analysis

**Fig. 2:**
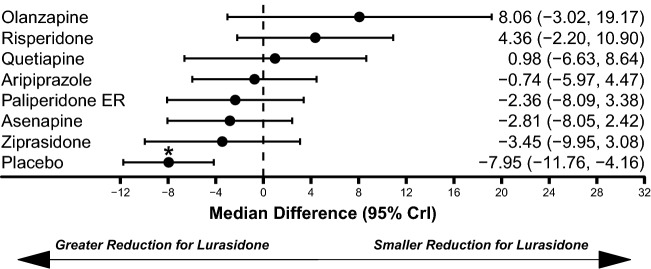
Comparison of PANSS Total Score Improvement for Lurasidone Relative to Comparators. Abbreviation: PANSS, Positive and Negative Syndrome Scale. *Statistically significant compared to lurasidone. Dashed line at 0 represents no difference from lurasidone

**Fig. 3:**
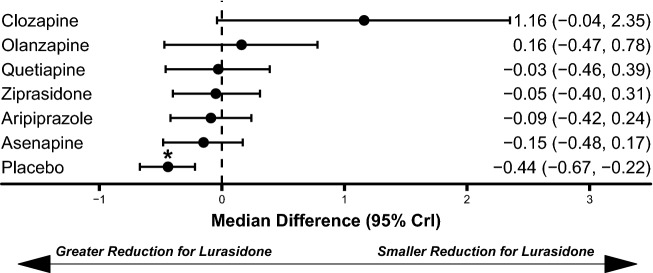
Comparison of CGI-S score improvement for lurasidone relative to comparators. *CGI-S* Clinical Global Impressions-Severity Scale. *Statistically significant compared to lurasidone. Dashed line at 0 represents no difference from lurasidone

Network meta-analysis results for the efficacy outcomes are presented in Figs. 2 and 3. Lurasidone demonstrated a statistically significantly greater improvement in PANSS score change from baseline compared to placebo (median difference: − 7.95, 95% CrI − 11.76 to − 4.16; Fig. 2). Similarly, lurasidone was associated with a greater improvement in CGI-S score change from baseline compared to placebo (median difference: − 0.44, 95% Crl − 0.67 to − 0.22; Fig. 3). There were no significant differences in PANSS or CGI-S score improvement between lurasidone and any comparator.

**Fig. 4:**
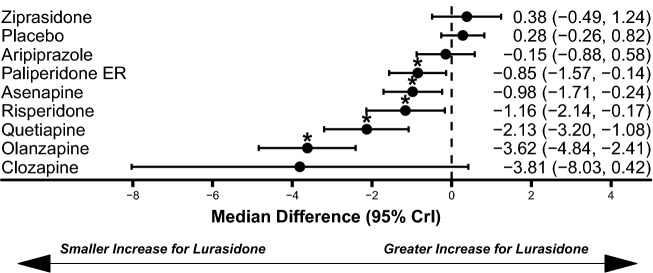
Comparison of change in body weight (kg) for lurasidone relative to comparators. *Statistically significant compared to lurasidone. Dashed line at 0 represents no difference from lurasidone

**Fig. 5:**
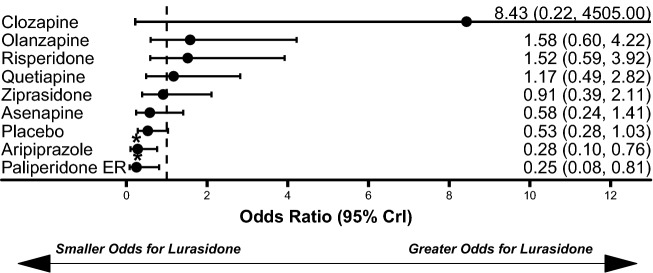
Comparisons of all-cause discontinuation for lurasidone relative to comparators. *Statistically significant compared to lurasidone. Dashed line at 1 represents no difference from lurasidone

**Fig. 6:**
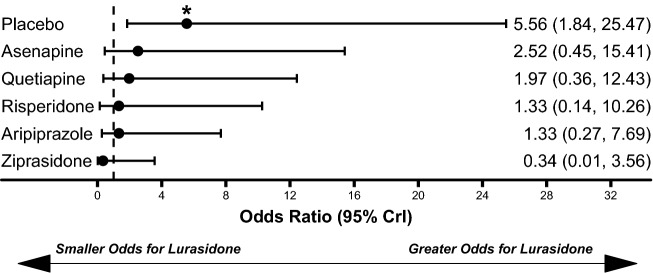
Comparison of extrapyramidal symptoms for lurasidone relative to comparators. *Statistically significant compared to lurasidone. Dashed line at 1 represents no difference from lurasidone

**Fig. 7:**
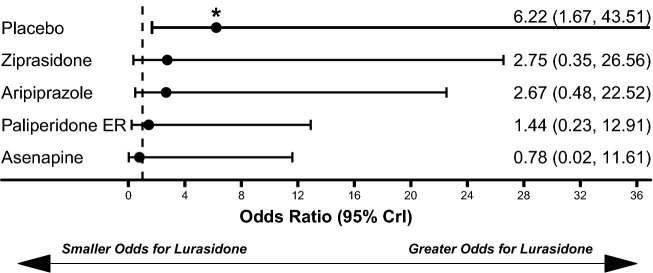
Comparison of Akathisia for lurasidone relative to comparators. *Statistically significant compared to lurasidone. Dashed line at 1 represents no difference from lurasidone

Lurasidone treatment was associated with similar weight gain to placebo (median difference: 0.26 kg, 95% CrI − 0.26 kg to 0.82 kg) and statistically significantly less weight gain compared to olanzapine (median difference: − 3.62 kg, 95% CrI − 4.84 kg to − 2.41 kg), quetiapine (median difference: − 2.13 kg, 95% CrI − 3.20 kg to − 1.08 kg), risperidone (median difference: − 1.16 kg, 95% CrI − 2.14 kg to − 0.17 kg), asenapine (median difference: − 0.98 kg, 95% CrI − 1.71 kg to − 0.24 kg), and paliperidone ER (median difference: − 0.85 kg, 95% CrI − 1.57 kg to − 0.14 kg; Fig. 4). Lurasidone treatment was also associated with significantly lower odds of all-cause discontinuations compared to aripiprazole (OR: 0.28, 95% CrI 0.10–0.76) and paliperidone ER (OR: 0.25, 95% CrI 0.08–0.81; Fig. 5). The odds of EPS (Fig. 6) and akathisia (Fig. 7) were not significantly different between lurasidone and comparators.

In addition to the primary results presented above, the analyses also examined several other outcome variables (see Online Appendix 2 for details). For response rate, the only additional efficacy measure, there were no significant differences between lurasidone and comparators. For the additional cardiometabolic tolerability variables, there were significantly greater increases in serum glucose and total cholesterol for lurasidone than ziprasidone and significantly smaller increases in triglycerides for lurasidone when compared to olanzapine. For somnolence and sedation, there were no significant differences between lurasidone and comparators. Finally, lurasidone had significantly fewer discontinuations due to adverse events compared to aripiprazole, asenapine, quetiapine, olanzapine, and paliperidone ER.

In addition, analyses compared all of the atypical antipsychotics to placebo (see Online Appendix 3 for details). While all of the antipsychotics were statistically significantly more efficacious than placebo, the tolerability profiles relative to placebo were varied. Relative to placebo, all of the antipsychotics were significantly more efficacious on PANSS score change from baseline (except for ziprasidone), CGI-S score change from baseline, and response rates (except for quetiapine). In terms of metabolic changes relative to placebo, there were significant increases in: body weight for paliperidone ER, asenapine, risperidone, quetiapine, and olanzapine; serum glucose for aripiprazole, asenapine, olanzapine, paliperidone ER, and ziprasidone (significantly decrease); total cholesterol for olanzapine, lurasidone, asenapine, paliperidone ER, and quetiapine; and triglycerides for quetiapine and olanzapine. There were significantly fewer all-cause discontinuations for olanzapine, risperidone, quetiapine, and ziprasidone and numerically fewer all-cause discontinuations for lurasidone, as compared with placebo. There were similar odds of discontinuations due to adverse events for all atypical antipsychotics as compared with placebo, with the exception of higher odds for olanzapine and paliperidone ER. There were significantly greater rates of EPS for aripiprazole, risperidone, lurasidone, and ziprasidone than placebo, as well as significantly greater rates of akathisia for paliperidone ER, lurasidone, and asenapine than placebo. All of the antipsychotics, except lurasidone and clozapine, had significantly higher rates of somnolence than placebo, and olanzapine and asenapine had significantly higher rates of sedation compared to placebo.

Finally, statistical comparisons between the atypical antipsychotics on the main outcome variables are presented in table form in Online Appendix 4. In terms of efficacy, there were few significant differences between antipsychotics, with risperidone having significantly greater reduction in PANSS total score than asenapine and ziprasidone, and clozapine having significantly greater reduction in CGI-S score than asenapine and aripiprazole. In terms of weight gain, there were multiple significant differences, with olanzapine having significantly greater weight gain than all other antipsychotics (except clozapine), and lurasidone, aripiprazole, and ziprasidone having significantly less weight gain than asenapine, paliperidone, quetiapine, and risperidone. Discontinuation rates were significantly lower for olanzapine, risperidone, quetiapine, and ziprasidone compared to aripiprazole and paliperidone, as well as for olanzapine and risperidone relative to asenapine. There were no significant differences between the antipsychotics in EPS or akathisia.

### Sensitivity analyses

Three sensitivity analyses were presented in Online Appendix 6. In the first sensitivity analysis, which involved removing two trials with a lower mean age at baseline (Mozes et al. [25], Shaw et al. [26]), lurasidone continued to be associated with statistically significantly less weight gain compared to olanzapine, quetiapine, asenapine and paliperidone ER, but was no longer statistically significantly better compared to risperidone.

The second sensitivity analysis removed trials of longer than 6 weeks duration. Removal of trials of 8- or 12-week duration did not result in changes in the conclusions regarding efficacy or tolerability in the base case findings. Lurasidone continued to be associated with significantly less weight gain compared to olanzapine and quetiapine, but it did not reach a statistically significant difference when compared with paliperidone ER or risperidone.

In the third sensitivity analysis, sequential removal of the two trials presenting direct evidence of olanzapine versus risperidone with conflicting direction on all-cause discontinuation (Mozes et al. [25], Jensen et al. [27]) did not alter any of the findings of the meta-analysis.

Conclusions from the random-effects models were the same as the fixed-effects models on all of the efficacy variables and for all of the placebo comparisons. A few comparisons between lurasidone and other antipsychotics on discontinuation rates and weight change that were just significant in the fixed-effects models were not significant in the random-effects models (see Online Appendix 5 for details).

## Discussion

This study compared the efficacy and tolerability of lurasidone to placebo and other atypical antipsychotics for the treatment of adolescents with schizophrenia. Lurasidone was significantly more efficacious as assessed by PANSS score and CGI-S score than placebo, and similarly efficacious when compared to other oral atypical antipsychotics in adolescents with schizophrenia. Adolescents treated with lurasidone showed statistically significantly less weight gain than adolescents treated with quetiapine, olanzapine, risperidone, asenapine, or paliperidone ER. Lurasidone was also associated with a lower risk of all-cause discontinuation compared to aripiprazole and paliperidone ER. There were no significant differences in the risk of EPS or akathisia between lurasidone and other comparators. These results suggest that lurasidone is an efficacious treatment for adolescent patients with schizophrenia with a lower risk of weight gain than most atypical antipsychotics.

Three other recent network meta-analyses compared antipsychotics in children or adolescents with schizophrenia (Pagsberg et al. [14], Harvey et al. [15], and Krause et al. [16]), but only one very recent study included lurasidone (Krause et al. [16]). Despite several important study design differences between the current study and Krause et al. conclusions regarding lurasidone were largely consistent. Krause et al. used broader study criteria and included studies of patients under 12 years of age, treatment with typical antipsychotics, and older studies using DSM II diagnostic criteria. These broad inclusion criteria allowed for 28 trials (rather than 12) to be incorporated. Krause et al. chose to measure efficacy using standardized mean change, combining multiple different reported efficacy measures, and used a random-effects model. All significant conclusions from the current study were consistent with a few exceptions: Krause et al. found statistically significantly greater efficacy for clozapine than lurasidone and did not find differences in discontinuation rates favoring lurasidone over paliperidone ER and aripiprazole. Both network meta-analyses found that lurasidone had a lower risk of weight gain compared to olanzapine, quetiapine, risperidone, and paliperidone [16].

As adolescents are particularly susceptible to atypical antipsychotic-induced weight gain, increased metabolic monitoring is urgently recommended for improving treatment outcomes [38–40]. Weight gain may decrease medication adherence [41, 42]. Overweight or obese adolescents may suffer from increased incidence of metabolic syndrome (38.1% in obese vs 1.5% in normal weight) and greater risk of developing hypertension [43]. In addition to the physical comorbidities associated with weight gain, psychological ramifications of weight gain in adolescence may include low self-esteem and depression [44, 45]. Some experts have suggested that atypical antipsychotics with a lower risk of cardiometabolic issues, including diabetes and weight gain, should be considered for first-line treatment for children with schizophrenia [16, 46].

## Limitations

A network meta-analysis can provide valuable information regarding the relative efficacy and tolerability of treatments that have not been directly compared, but this methodology is not without limitations. As with all meta-analyses, comparable populations and consistently defined outcomes across trials are assumed. Among the trials included in the present analysis, observable patient characteristics largely appeared similar, and where there were differences (such as age), sensitivity analyses were conducted to examine their potential impact on the main analysis. There were differences across trials where sensitivity analyses were not conducted, such as prior exposure to atypical antipsychotics and the reporting of EPS. The definitions of EPS varied across trials with some reporting any movement disorder, some separating akathisia from EPS, some only reporting changes in ratings scales, and some reporting rates of anticholinergic medication use. The potential effect of these differences on the results is unclear.

Although the number of included trials was sufficient to conduct a robust analysis, some trials had small sample sizes. This resulted in imprecise estimation of certain treatment effects for some comparators, such as clozapine. Our study examined only atypical antipsychotic monotherapy and did not include trials of adjunctive therapy. One trial was excluded [47] as its primary objective was to compare the typical antipsychotic molindone with olanzapine and risperidone; however, efficacy findings were similar with the results of our study. Finally, trials such as those included in this network meta-analysis are generally not powered to detect statistically significant differences between antipsychotics in the context of an indirect comparison. Therefore, the absence of a statistically significant difference does not indicate that a difference between treatments does not exist, but rather that a significant difference could not be detected based on the current evidence base, modeling methods, and assumptions used.

## Conclusions

The results of this network meta-analysis suggest lurasidone has comparable efficacy to other atypical antipsychotics for adolescent patients with schizophrenia-spectrum disorders. The tolerability profile of lurasidone was relatively favorable with a lower risk of weight gain compared to asenapine, olanzapine, risperidone, paliperidone ER, and quetiapine. The observed rates of EPS or akathisia were comparable across atypical antipsychotics. Treatments for schizophrenia that effectively balance efficacy and tolerability in adolescents may result in better patient health outcomes.

## Electronic supplementary material

Below is the link to the electronic supplementary material.

Supplementary material 1 (PDF 122 kb)

Supplementary material 2 (PDF 226 kb)

Supplementary material 3 (PDF 379 kb)

Supplementary material 4 (PDF 76 kb)

Supplementary material 5 (PDF 71 kb)

Supplementary material 6 (PDF 112 kb)
