# Supplementary material for: Lurasidone compared to other atypical antipsychotic monotherapies for adolescent schizophrenia: a systematic literature review and network meta-analysis
Source: Eur Child Adolesc Psychiatry. 2019 Nov 22;29(9):1195–205. doi: 10.1007/s00787-019-01425-2 (PMC7497364; doi:10.1007/s00787-019-01425-2)
Supplement: Supplementary file 1 — Supplementary material 1 (PDF 122 kb) [file 787_2019_1425_MOESM1_ESM.pdf]

Appendix 1. Systematic Literature Review

The systematic literature review followed methods outlined in guidelines published by the Cochrane Collaboration [1] and National Institute for Health and Care Excellence (NICE) [2]. NICE has a rigorous and clearly specified approach to systematic literature reviews. A review conducted to a standard suitable for NICE can be considered sufficient by health technology assessment agencies in other countries.

The scope of the search strategy was defined in terms of Population, Intervention, Comparison, Outcome Measures, and Study Design (PICOS) criteria. The PICOS statement is listed in Table 1.

The OvidSP platform was used to conduct the searches. This is a search platform that provides standardized access to a wide range of clinical literature databases and is an accepted tool for use in systematic literature reviews. The following databases were searched to identify relevant published studies:

- Embase (1988 to 2016 Week 13)
- Ovid MEDLINE® In Process & Other Non-Indexed Citations and Ovid MEDLINE® (1946 to present)
- Cochrane Library: EBM Reviews – Database of Abstracts of Reviews of Effects (DARE) (1991 to 1st Quarter)

The search strategy followed the structure outlined in Table 2.

The studies were reviewed based on specified inclusion and exclusion criteria. These criteria aligned with PICOS and are listed in Table 3. The specific search terms used and number of articles identified by each search term in each database are listed

in Tables 4, 5, and 6. Twenty-five full text articles were assessed for eligibility. Table 7 lists the 13 studies [3-15] that were excluded after reviewing the full text articles and details the specific reasons for exclusion. Figure 1 presents a graphical outline of the final network of evidence without taking into account the availability of specific outcome measures.

References

1. Higgins JPT Green S (2011) Cochrane handbook for systematic reviews of interventions version 5.1.0. http://handbook-5-1.cochrane.org. Accessed 1 February 2019

2. Dias S, Welton NJ, Sutton AJ, Ades A (2011) NICE DSU technical support document 2: A generalised linear modelling framework for pairwise and network meta-analysis of randomised controlled trials. NICE Decision Support Unit. https://www.ncbi.nlm.nih.gov/books/NBK310366/pdf/Bookshelf_NBK310366.pdf. Accessed 1 February 2019

3. Findling RL, Cavus I, Pappadolulos E, Backinsky M, Schwartz JH, Vanderburg DG (2010) A placebo-controlled trial to evaluate the efficacy and safety of flexibly dosed oral ziprasidone in adolescent subjects with schizophrenia. 2nd Biennial Schizophrenia International Research Society Conference, Florence Italy April 14 2010. Schizophr Res 117:437

4. Robinson DG, Gallego JA, John M, Petrides G, Hassoun Y, Zhang J-P, et al (2015) A randomized comparison of aripiprazole and risperidone for the acute treatment of first-episode schizophrenia and related disorders: 3-month outcomes. Schizophr Bull 41:1227-1236

5. Kemp DE, Correll CU, Tohen M, DelBello MP, Ganocy SJ, Findling RL, et al (2013) Associations among obesity, acute weight gain, and response to treatment with olanzapine in adolescent schizophrenia. J Child Adolesc Psychopharmacol 23:522-530

6. Huffman GB (1997) Efficacy of clozapine for schizophrenia in children. American Family Physician 55:1356

7. Kumra S. Is there a role for clozapine in children and adolescents with schizophrenia? 2nd Biennial Schizophrenia International Research Society Conference, Florence Italy April 14 2010. Schizophr Res 117:166

8. McCormack PL (2010) Olanzapine: In adolescents with schizophrenia or bipolar I disorder. CNS Drugs 24: 443-452

9. Whitehead RE, Wisneiwski SR, Ali M, Jin N, Kim E, Nyilas M, et al (2009) Patient assessed quality of life versus clinician assessment: A post-hoc analysis of a trial of aripiprazole in adolescent patients with schizophrenia. The 49th Annual National Institute of Mental Health (NIMH) New Clinical Drug Evaluation Unit (NCDEU) Meeting, Hollywood, Florida, June 29 2009. J Child Adolesc Psychopharmacol 19(6):1

10. Mathew S, Pikalov A, McQuade RD, Carson WH, Nyilas M, Forbes RA, et al (2009) Somnolence and sedation in adolescent patients with schizophrenia treated with aripiprazole in an acute study with long-term follow-up. The 49th Annual National Institute of Mental Health (NIMH) New Clinical Drug Evaluation Unit (NCDEU) Meeting, Hollywood, Florida, June 29 2009. J Child Adolesc Psychopharmacol 19(6):1

11. Kryzhanovskaya LA, Robertson-Plouch CK, Xu W, Carlson JL, Merida KM, Dittmann RW (2009) The safety of olanzapine in adolescents with schizophrenia or bipolar I disorder: a pooled analysis of 4 clinical trials. J Clin Psychiatry 70:247-258

12. Correll CU. Time course and relevance of early treatment response in adolescents with schizophrenia-spectrum disorders. 2nd Biennial Schizophrenia International Research Society Conference, Florence Italy April 14 2010. Schizophr Res 117:131-132

13. Gallego JA, Robinson DG, Sevy SM, Napolitano B, McCormack J, Lesser ML, et al (2011) Time to treatment response in first-episode schizophrenia: should acute treatment trials last several months? J Clin Psychiatry 72:1691-1696

14. Correll C, Murray S, Zhao J, Manos G, Mankoski R, Forbes R, et al (2010) Weight gain and metabolic changes in studies of aripiprazole for the treatment of pediatric bipolar I disorder and adolescent schizophrenia. 49th Annual Conference of the American College of Neuropsychopharmacology, Miami Beach, FL United States December 4 2010. Neuropsychopharmacology 35(Suppl 1)S387-388

15. Correll CU, Kohegyi E, Zhao C, Baker RA, McQuade R, Salzman P, et al (2014) Oral aripiprazole is an effective maintenance treatment in adolescents with schizophrenia: A randomized, double-blind, placebo-controlled trial. 53rd Annual Conference of the American College of Neuropsychopharmacology, Phoenix, AZ United States, December 7 2014. Neuropsychopharmacology 39(Suppl 1): S349-S350

Table 1. PICOS statement

PICOS	Scope
Patient Population	Humans 12-17 years of age with schizophrenia, including subsets of this age range
Intervention and Comparisons	Interventions: Lurasidone, aripiprazole, asenapine, clozapine, olanzapine, paliperidone, risperidone, quetiapine and ziprasidone<br>Comparisons: Placebo or any of the above interventions
Outcome Measures	1. Change from baseline in CGI-S scale<br>2. Change from baseline in PANSS total score<br>3. Response<br>4. Relapse<br>5. Change from baseline in weight<br>6. Change from baseline in Body Mass Index (BMI)<br>7. Change from baseline in fasting high-density lipoprotein (HDL) cholesterol<br>8. Change from baseline in fasting low-density lipoprotein (LDL) cholesterol<br>9. Change from baseline in fasting total cholesterol<br>10. Change from baseline in fasting triglycerides<br>11. Change from baseline in fasting serum glucose<br>12. Discontinuation due to all causes<br>13. Discontinuation due to lack of efficacy<br>14. Discontinuation due to adverse events (AEs)<br>15. Akathisia<br>16. Extrapyramidal symptoms (EPS)<br>17. Sedation<br>18. Somnolence
Study Design	Randomized controlled trials (RCTs) (including head-to-head and placebo-controlled trials)
Restrictions	Language: English

Table 2: Structure of the Search Strategy

Clinical Evaluations
Disease terms
**AND**
Intervention terms
**AND**
Study Design terms
**AND**
Population terms

Table 3. Inclusion and Exclusion Criteria

	Inclusion Criteria	Exclusion Criteria
Patient Population	Humans 12-17 years of age with schizophrenia, including subsets of this age range	Non-humans, non-schizophrenic patients, patients not within this age range or a subset
Interventions	• Lurasidone<br>• Aripiprazole<br>• Asenapine<br>• Clozapine<br>• Olanzapine<br>• Paliperidone<br>• Risperidone<br>• Quetiapine<br>• Ziprasidone	Any treatments other than those listed in inclusion criteria
Comparators	Any of the above listed interventions and placebo	Comparators other than those listed in the inclusion criteria
Outcome Measures	• Change from baseline in CGI-S scale<br>• Change from baseline in PANSS total score<br>• Response<br>• Relapse<br>• Change from baseline in weight<br>• Change from baseline in Body Mass Index (BMI)<br>• Change from baseline in fasting high-density lipoprotein (HDL) cholesterol<br>• Change from baseline in fasting low-density lipoprotein (LDL) cholesterol<br>• Change from baseline in fasting total cholesterol<br>• Change from baseline in fasting triglycerides<br>• Change from baseline in fasting serum glucose<br>• Discontinuation due to all causes<br>• Discontinuation due to lack of efficacy<br>• Discontinuation due to adverse events (AEs)<br>• Akathisia<br>• Extrapyramidal symptoms (EPS)<br>• Sedation<br>• Somnolence	Outcome measures other than those listed in the inclusion criteria
Study design	Randomized controlled trials (RCTs) (including head-to-head and placebo-controlled trials)	Non-randomized controlled trials
Restrictions	English language studies	Non-English language studies

Table 4. Clinical Search Terms - Embase

#	Search String	Hits
1	exp schizophrenia/	129,856
2	schizophrenia.mp.	141,500
3	1 or 2	144,306
4	lurasidone.mp.	838
5	aripiprazole.mp.	11,225
6	asenapine.mp.	986
7	clozapine.mp.	27,390
8	olanzapine.mp.	28,533
9	paliperidone.mp.	2,833
10	risperidone.mp.	30,855
11	quetiapine.mp.	18,771
12	ziprasidone.mp.	7,854
13	4 or 5 or 6 or 7 or 8 or 9 or 10 or 11 or 12	65,474
14	juvenile.mp.	87,621
15	childhood onset.mp.	5,444
16	young adult.mp.	146,614
17	child*.mp.	1,702,124
18	schoolchild*.mp.	10,749
19	school age*.mp.	18,396
20	kid*.mp.	799,600
21	adoles*.mp.	1,112,806
22	teen*.mp.	28,032
23	boy*.mp.	140,981
24	girl*.mp.	134,061
25	minors*.mp.	2,952

#	Search String	Hits
26	pubert*.mp.	38,571
27	paediatric*.mp.	73,920
28	pediatric*.mp.	344,266
29	schools.mp.	62,016
30	primary school*.mp.	14,166
31	secondary school*.mp.	8,162
32	elementary school*.mp.	7,730
33	high school*.mp.	30,802
34	14 or 15 or 16 or 17 or 18 or 19 or 20 or 21 or 22 or 23 or 24 or 25 or 26 or 27 or 28 or 29 or 30 or 31 or 32 or 33	3,210,595
35	random*.tw. or placebo*.mp. or double-blind*.tw.	1,193,846
36	3 and 13 and 34 and 35	623

Note. exp – explode search term; .mp. – multiple field search; .tw. - text word in title or abstract, .pt. - publication type; .adj. – adjacent to; ? – wildcard for 0 or 1 characters; * - wildcard multiple characters.

Table 5. Clinical Search Terms - MEDLINE

#	Search String	Hits
1	schizophrenia.mp.	117,848
2	lurasidone.mp.	220
3	aripiprazole.mp.	2,913
4	asenapine.mp.	270
5	clozapine.mp.	10,644
6	olanzapine.mp.	7,542
7	paliperidone.mp.	801
8	risperidone.mp.	8,299
9	quetiapine.mp.	3,959
10	ziprasidone.mp.	1,721
11	2 or 3 or 4 or 5 or 6 or 7 or 8 or 9 or 10	25,099
12	juvenile.mp.	68,455
13	childhood onset.mp.	3,772
14	young adult.mp.	502,135
15	child*.mp.	2,033,496
16	schoolchild*.mp.	11,373
17	school age*.mp.	16,163
18	kid*.mp.	736,713
19	adoles*.mp.	1,756,425
20	teen*.mp.	23,985
21	boy*.mp.	123,533
22	girl*.mp.	118,165
23	minors*.mp.	4,789
24	pubert*.mp.	36,927
25	paediatric*.mp.	46,554

26	pediatric*.mp.	248,016
27	schools.mp.	109,178
28	primary school*.mp.	8,835
29	secondary school*.mp.	7,498
30	elementary school*.mp.	7,363
31	high school*.mp.	22,909
32	12 or 13 or 14 or 15 or 16 or 17 or 18 or 19 or 20 or 21 or 22 or 23 or 24 or 25 or 26 or 27 or 28 or 29 or 30 or 31	3,984,468
33	randomized controlled trial.pt.	410,684
34	(random* or double-blind*).tw or placebo*.mp	910,128
35	33 or 34	995,219
36	1 and 11 and 32 and 35	692

Note. exp – explode search term; .mp. – multiple field search; .tw. - text word in title or abstract, .pt. - publication type; .adj. – adjacent to; ? – wildcard for 0 or 1 characters; * - wildcard multiple characters.

Table 6. Clinical Search Terms - Cochrane Library

#	Search String	Hits
1	schizophrenia.mp.	402
2	lurasidone.mp.	2
3	aripiprazole.mp.	56
4	asenapine.mp.	8
5	clozapine.mp.	88
6	olanzapine.mp.	127
7	paliperidone.mp.	12
8	risperidone.mp.	139
9	quetiapine.mp.	84
10	ziprasidone.mp.	45
11	2 or 3 or 4 or 5 or 6 or 7 or 8 or 9 or 10	227
12	randomi?ed control* trial*.tw.	15,107
13	clinical trial*.mp.	4,041
14	random*.mp.	17,428
15	(doubl* adj blind*).mp.	2,266
16	placebo*.tw. or placebo.mp.	4,128
17	(singl* adj blind*).mp.	295
18	assign*.mp.	36,632
19	allocate*.mp.	371
20	rct.tw.	2,887
21	12 or 13 or 14 or 15 or 16 or 17 or 18 or 19 or 20	36,632
22	juvenile.mp.	63
23	childhood onset.mp.	5
24	young adult.mp.	630
25	child*.mp.	3,834

26	schoolchild*.mp.	17
27	school age*.mp.	89
28	kid*.mp.	906
29	adoles*.mp.	2,218
30	teen*.mp.	56
31	boy*.mp.	96
32	girl*.mp.	96
33	minors*.mp.	18
34	pubert*.mp.	15
35	paediatric*.mp.	558
36	pediatric*.mp.	343
37	schools.mp.	268
38	primary school*.mp.	32
39	secondary school*.mp.	28
40	elementary school*.mp.	17
41	high school*.mp.	44
42	22 or 23 or 24 or 25 or 26 or 27 or 28 or 29 or 30 or 31 or 32 or 33 or 34 or 35 or 36 or 37 or 38 or 39 or 40 or 41	5,982
43	1 and 11 and 21 and 42	22

Note. exp – explode search term; .mp. – multiple field search; .tw. - text word in title or abstract, .pt. - publication type; .adj. – adjacent to; ? – wildcard for 0 or 1 characters; * - wildcard multiple characters.

Table 7. List of Excluded Studies and Reasons for Exclusion

#	Author and year	Reason for exclusion	Reference
1	Findling et al., 2010	Conference abstract. Full paper included in review.	[3]
2	Robinson et al., 2015	Incorrect population. 15-40 year olds with no subgroup results.	[4]
3	Kemp et al., 2013	Post-hoc analysis of a full paper included in review.	[5]
4	Huffman, 1997	Incorrect comparator. First generation antipsychotic.	[6]
5	Kumra, 2010	Limited outcome data as only abstract was available.	[7]
6	McCormack, 2010	Full text review revealed this to be a review.	[8]
7	Whitehead et al., 2009	Post-hoc analysis of a full paper included in review.	[9]
8	Mathew et al., 2009	Post-hoc analysis of a full paper included in review.	[10]
9	Kryzhanovskaya et al., 2009	Incorrect population. Pooled analysis of schizophrenia and bipolar trials.	[11]
10	Correll, 2010	Irrelevant outcomes. Examined impact of early response.	[12]
11	Gallego et al., 2011	Incorrect population. 16-40 year olds with no subgroup results.	[13]
12	Correll et al., 2010	Irrelevant outcomes reported.	[14]
13	Correll et al., 2014	No study duration in trial protocol. Patients were all discontinued after 37 relapse events.	[15]

Figure 1. Network of Evidence

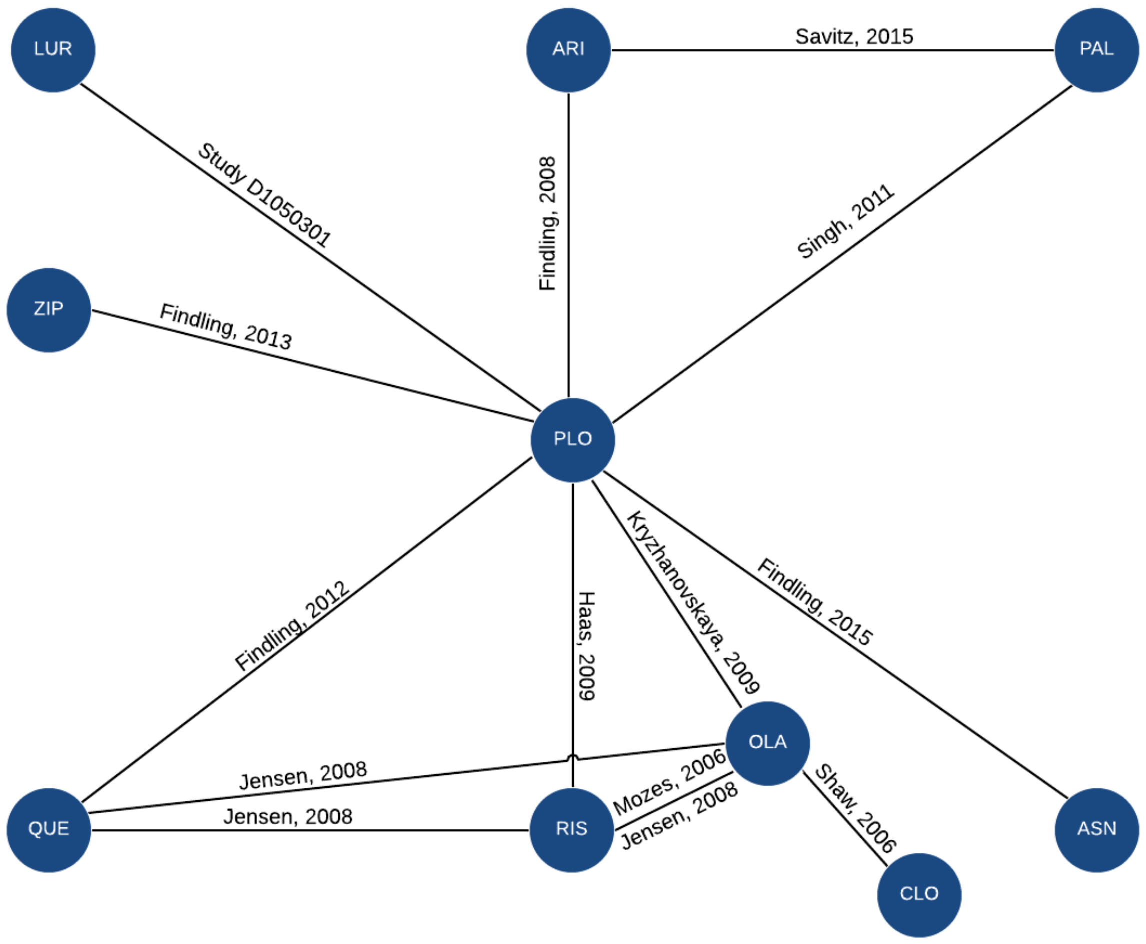

Abbreviations: ARI aripiprazole; ASN asenapine; CLO clozapine; LUR lurasidone; OLA olanzapine; PAL paliperidone extended-release; PLO placebo; QUE quetiapine; RIS risperidone; ZIP ziprasidone
