# Supplementary material for: Lurasidone compared to other atypical antipsychotic monotherapies for adolescent schizophrenia: a systematic literature review and network meta-analysis
Source: Eur Child Adolesc Psychiatry. 2019 Nov 22;29(9):1195–205. doi: 10.1007/s00787-019-01425-2 (PMC7497364; doi:10.1007/s00787-019-01425-2)
Supplement: Supplementary file 2 — Supplementary material 2 (PDF 226 kb) [file 787_2019_1425_MOESM2_ESM.pdf]

Appendix 2. Network Meta-Analysis of Additional Outcome Variables

Fig 1. Forest Plot of Response Rates: Lurasidone Versus Comparators

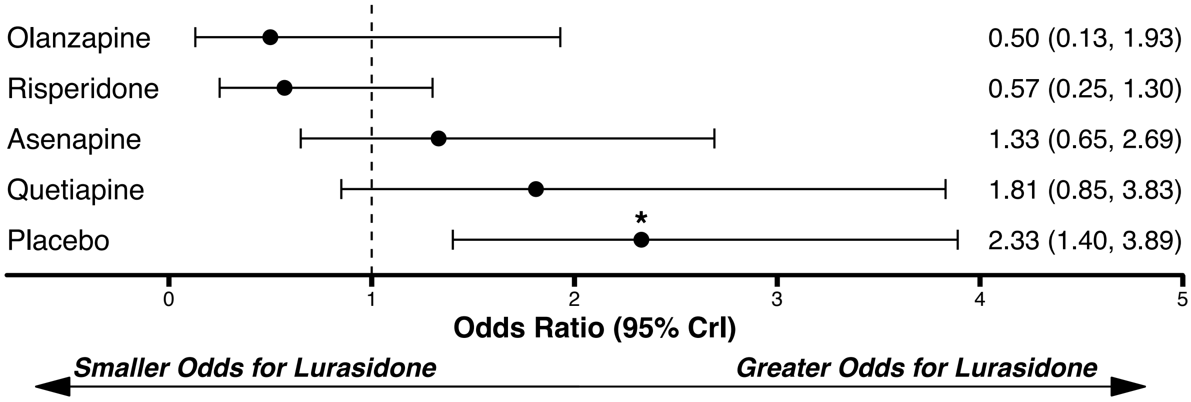

* Statistically significant compared to lurasidone

Dashed line at 1 represents no difference from lurasidone.

Response was defined as a ≥20% improvement in PANSS total score with 3 exceptions: Mozes 2006 and Findling 2012 reported it as ≥30% improvement in PANSS total score and Jensen 2008 reported it as ≥40% improvement in PANSS total score.

Fig 2. Forest Plot of Somnolence: Lurasidone vs. Comparators

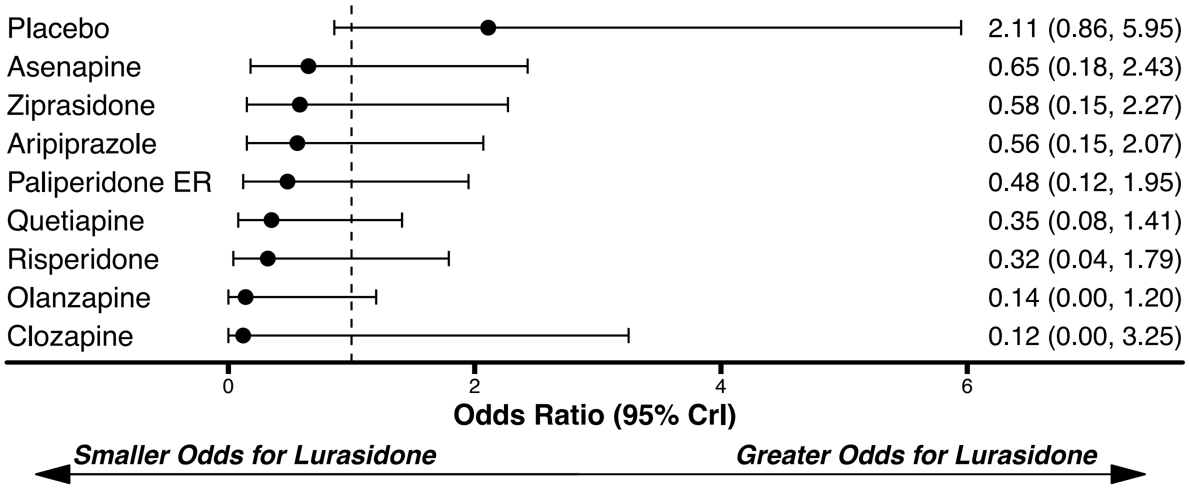

* Statistically significant compared to lurasidone

Dashed line at 1 represents no difference from lurasidone.

Fig 3. Forest Plot of Sedation: Lurasidone versus Comparators

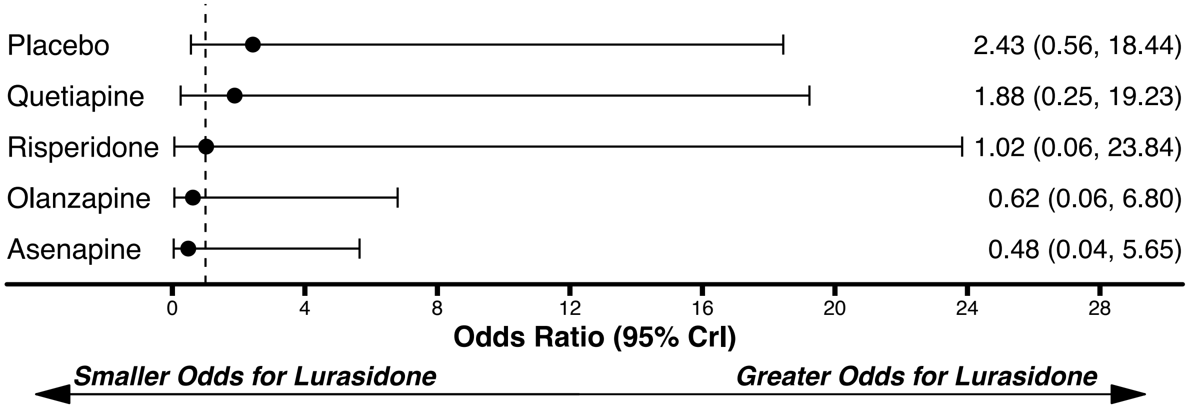

* Statistically significant compared to lurasidone

Dashed line at 1 represents no difference from lurasidone.

Fig 4. Forest Plot of Change from Baseline in Serum Glucose: Lurasidone vs. Comparators

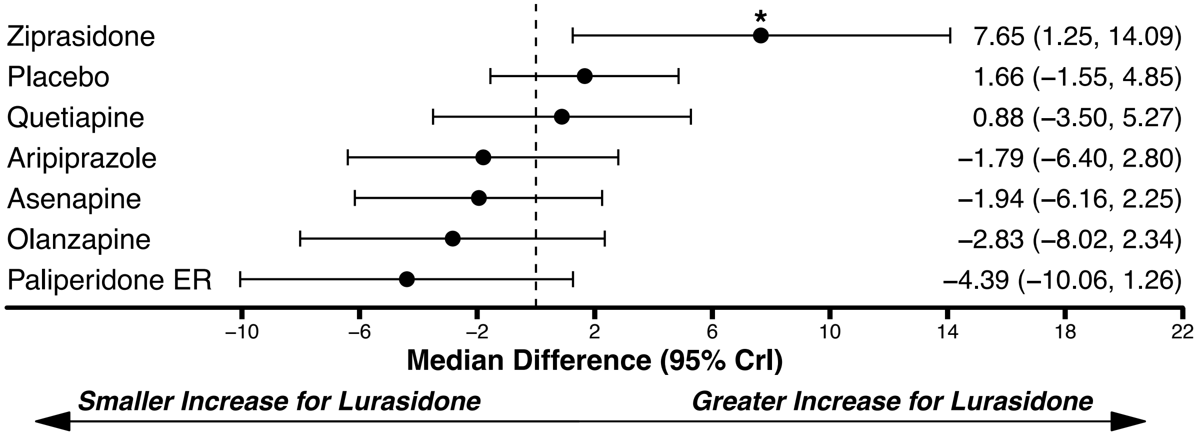

* Statistically significant compared to lurasidone

Dashed line at 0 represents no difference from lurasidone.

Fig 5. Forest Plot of Change from Baseline in Total Cholesterol: Lurasidone vs. Comparators

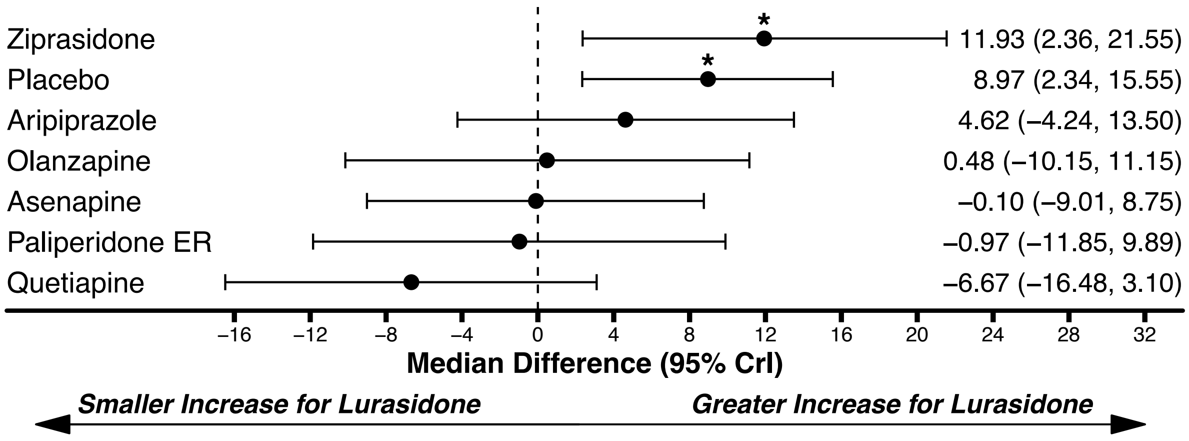

* Statistically significant compared to lurasidone

Dashed line at 0 represents no difference from lurasidone.

Fig 6. Forest Plot of Change from Baseline in Serum Triglycerides: Lurasidone vs. Comparators

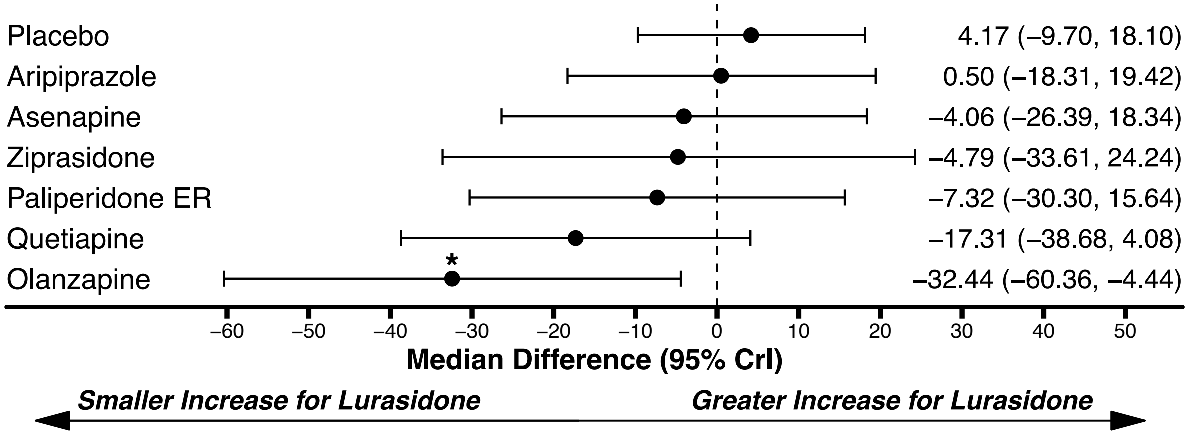

* Statistically significant compared to lurasidone

Dashed line at 0 represents no difference from lurasidone.

Fig 7. Forest Plot of Discontinuation Due to Adverse Events: Lurasidone vs. Comparators

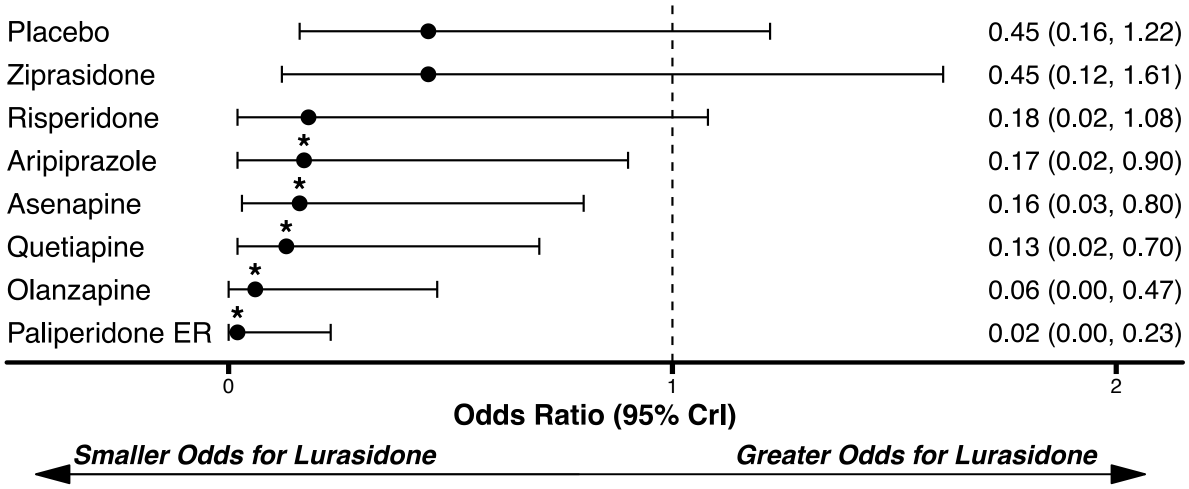

* Statistically significant compared to lurasidone

Dashed line at 1 represents no difference from lurasidone.
