# Supplementary material for: Lurasidone compared to other atypical antipsychotic monotherapies for adolescent schizophrenia: a systematic literature review and network meta-analysis
Source: Eur Child Adolesc Psychiatry. 2019 Nov 22;29(9):1195–205. doi: 10.1007/s00787-019-01425-2 (PMC7497364; doi:10.1007/s00787-019-01425-2)
Supplement: Supplementary file 3 — Supplementary material 3 (PDF 379 kb) [file 787_2019_1425_MOESM3_ESM.pdf]

Appendix 3. Comparisons with Placebo

Fig 1. Forest Plot of Change from Baseline in PANSS Total Score Compared to Placebo

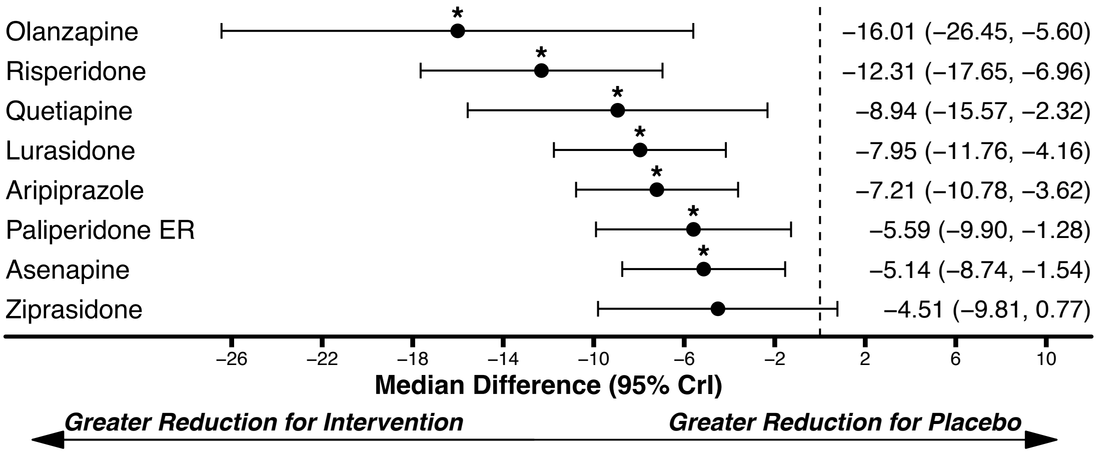

* Statistically significant compared to placebo

Dashed line at 0 represents no difference from placebo.

Fig 2. Forest Plot of Change from Baseline in CGI-S Score Compared to Placebo

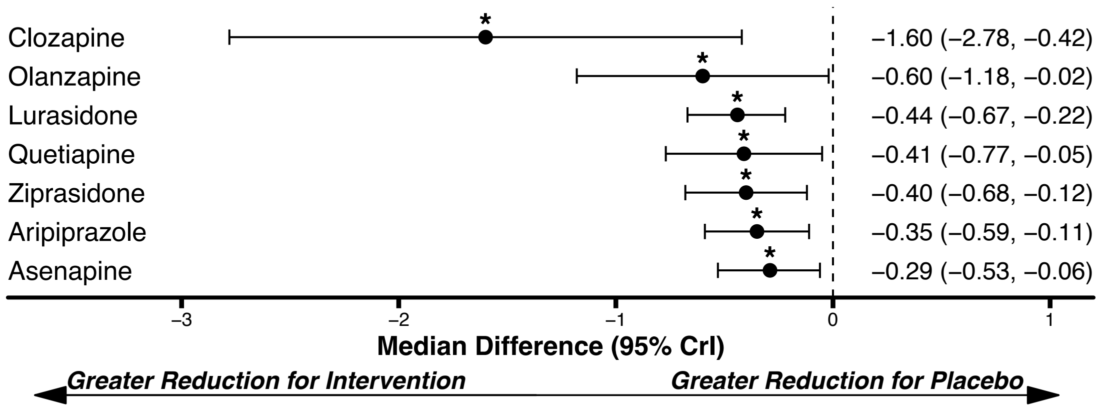

* Statistically significant compared to placebo

Dashed line at 0 represents no difference from placebo.

Fig 3. Forest Plot of Response Rates Compared to Placebo

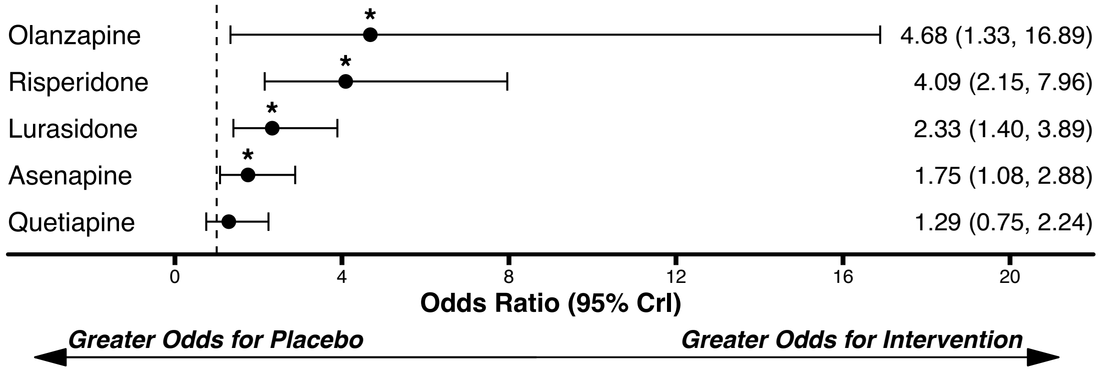

* Statistically significant compared to placebo

Dashed line at 1 represents no difference from placebo.

Response was defined as a ≥20% improvement in PANSS total score with 3 exceptions: Mozes 2006 and Findling 2012 reported it as ≥30% improvement in PANSS total score and Jensen 2008 reported it as ≥40% improvement in PANSS total score.

Fig 4. Forest Plot of Change from Baseline in Body Weight Compared to Placebo

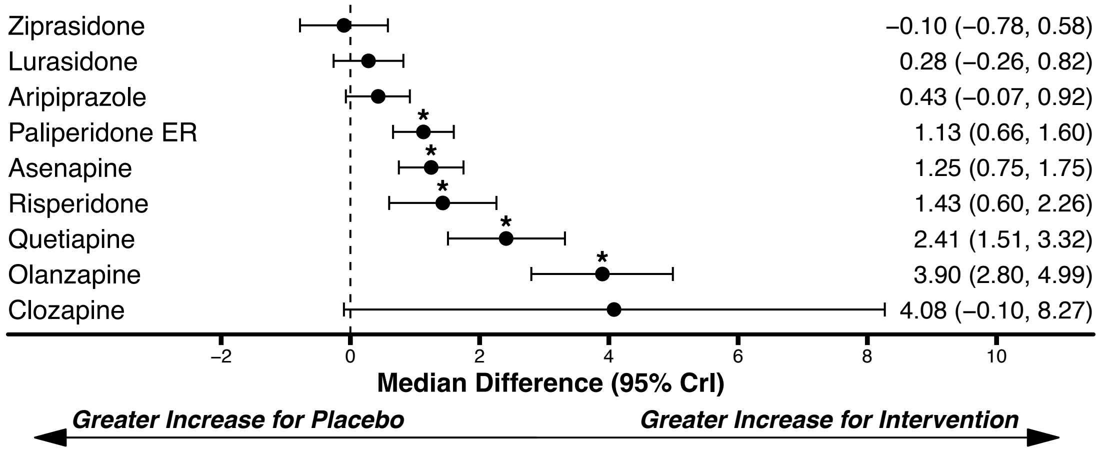

* Statistically significant compared to placebo

Dashed line at 0 represents no difference from placebo.

Fig 5. Forest Plot of Change from Baseline in Serum Glucose Compared to Placebo

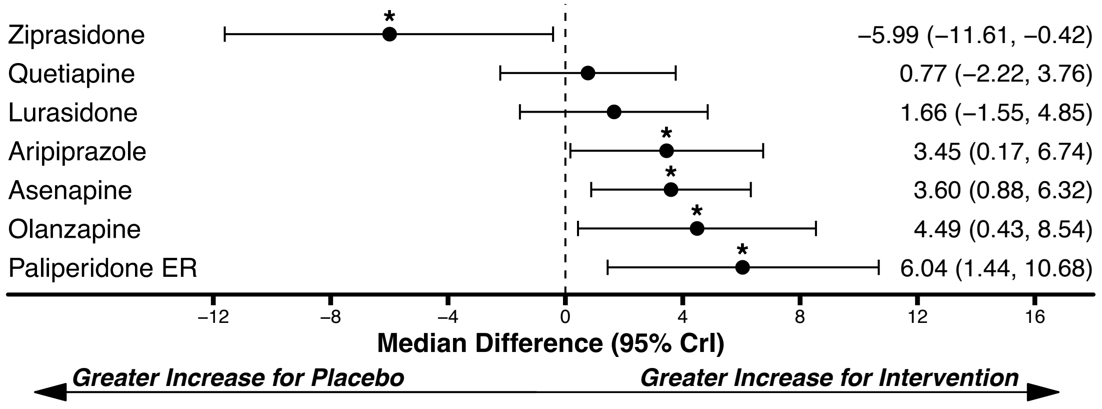

* Statistically significant compared to placebo

Dashed line at 0 represents no difference from placebo.

Fig 6. Forest Plot of Change from Baseline in Total Cholesterol Compared to Placebo

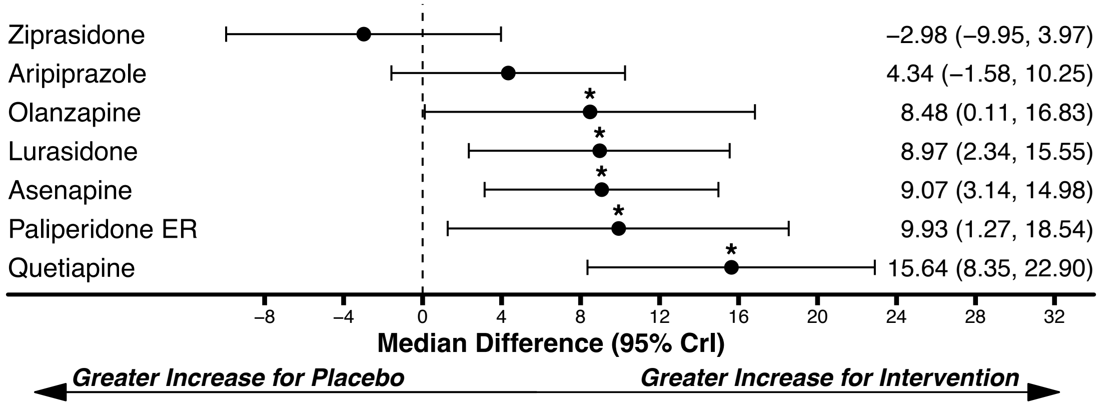

* Statistically significant compared to placebo

Dashed line at 0 represents no difference from placebo.

Fig 7. Forest Plot of Change from Baseline in Triglycerides Compared to Placebo

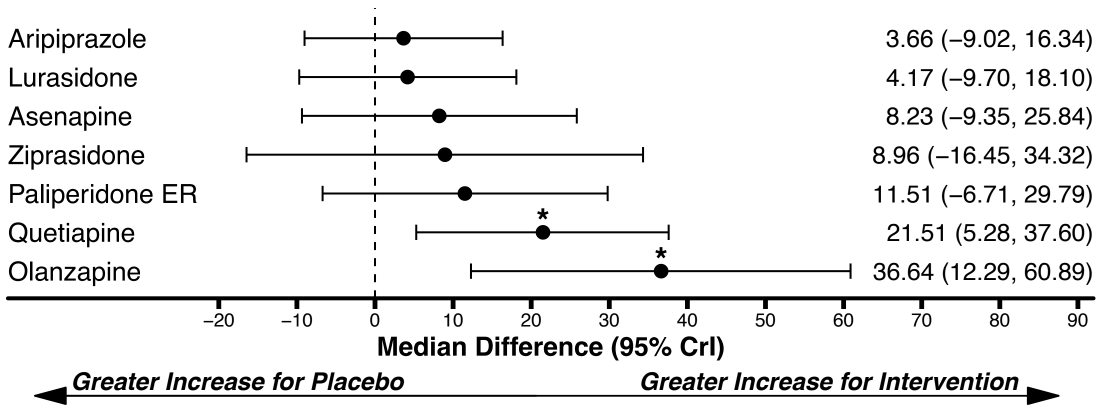

* Statistically significant compared to placebo

Dashed line at 0 represents no difference from placebo.

Fig 8. Forest Plot of All-Cause Discontinuation Compared to Placebo

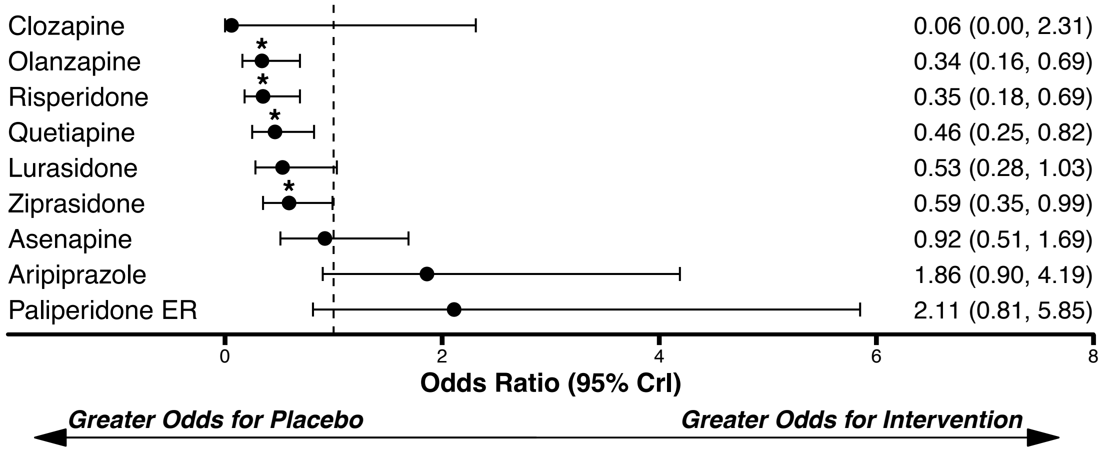

* Statistically significant compared to placebo

Dashed line at 1 represents no difference from placebo.

Fig 9. Forest Plot of Discontinuation Due to Adverse Events Compared to Placebo

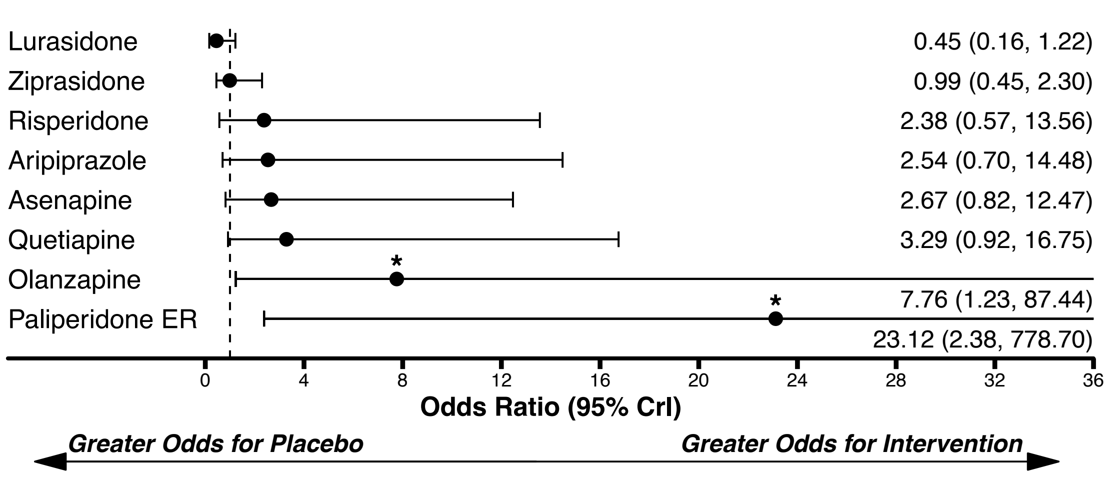

* Statistically significant compared to placebo

Dashed line at 1 represents no difference from placebo.

Fig 10. Forest Plot of Extrapyramidal Symptoms Compared to Placebo

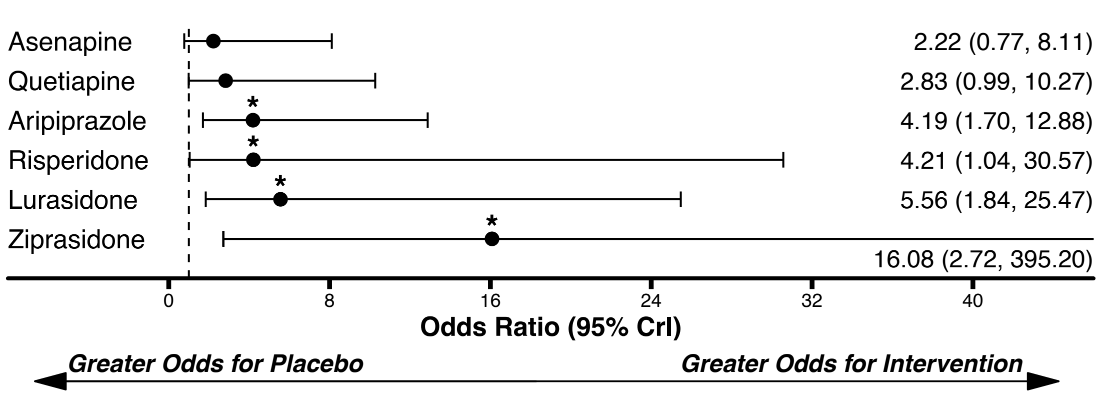

* Statistically significant compared to placebo

Dashed line at 1 represents no difference from placebo.

Fig 11. Forest Plot of Akathisia Compared to Placebo

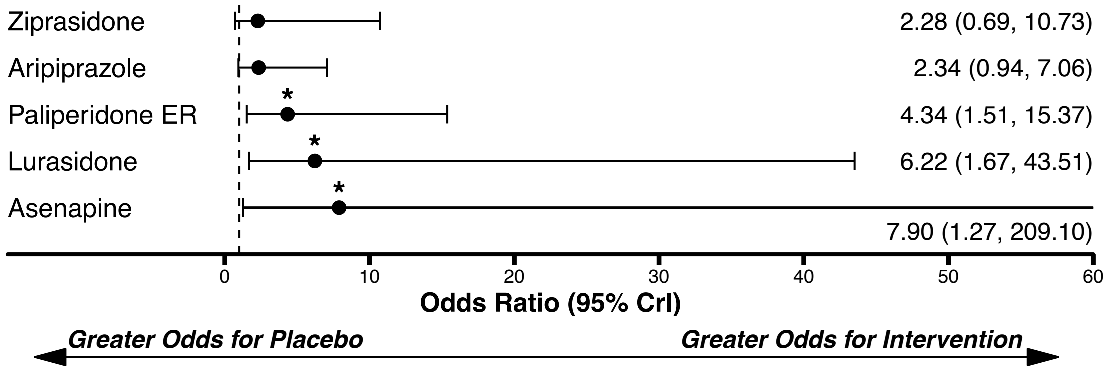

* Statistically significant compared to placebo

Dashed line at 1 represents no difference from placebo.

Fig 12. Forest Plot of Somnolence Compared to Placebo

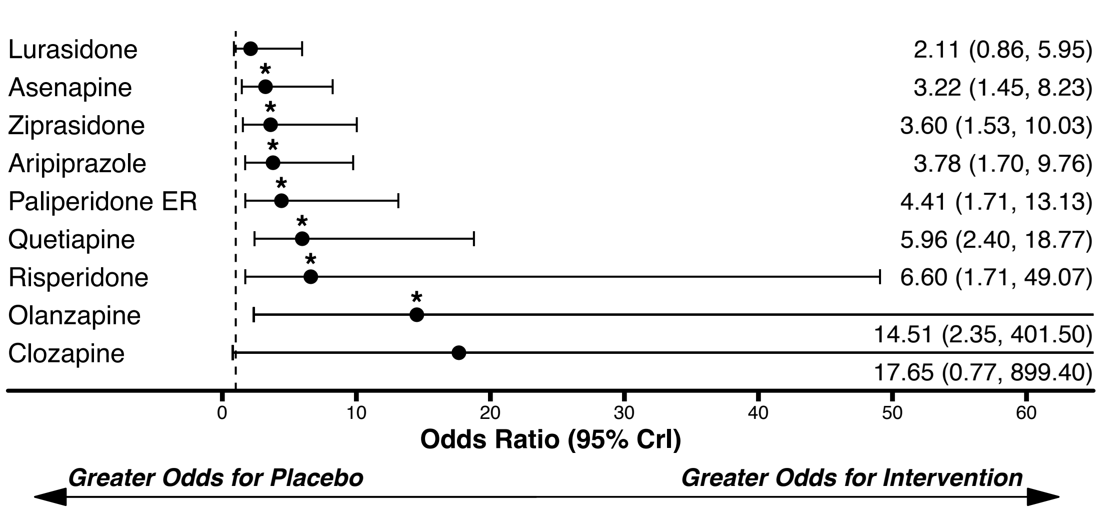

* Statistically significant compared to placebo

Dashed line at 1 represents no difference from placebo.

Fig 13. Forest Plot of Sedation Compared to Placebo

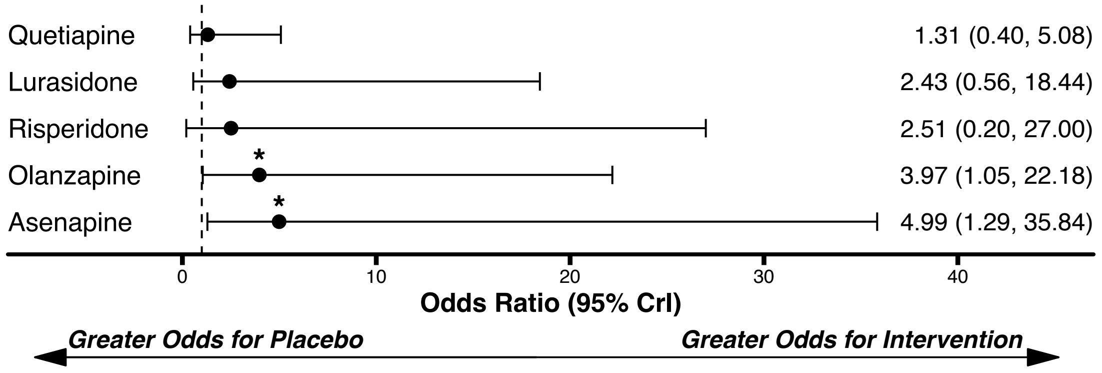

* Statistically significant compared to placebo

Dashed line at 1 represents no difference from placebo.
