# Supplementary material for: Lurasidone compared to other atypical antipsychotic monotherapies for adolescent schizophrenia: a systematic literature review and network meta-analysis
Source: Eur Child Adolesc Psychiatry. 2019 Nov 22;29(9):1195–205. doi: 10.1007/s00787-019-01425-2 (PMC7497364; doi:10.1007/s00787-019-01425-2)
Supplement: Supplementary file 4 — Supplementary material 4 (PDF 76 kb) [file 787_2019_1425_MOESM4_ESM.pdf]

Appendix 4. Comparisons Between All Treatments

The results parallel to those reported in the main manuscript that compare all of the atypical antipsychotics are reported in the tables of this appendix. The main outcome variables include change in PANSS Total score (Table 1), change in CGI-S score (Table 1), change in body weight (Table 2), rate of all-cause discontinuation (Table 2), rate of extrapyramidal symptoms (EPS, Table 3), and rate of akathisia (Table 3).

On the PANSS Total score, all of the atypical antipsychotics (except ziprasidone) were significantly more efficacious than placebo and risperidone was significantly more efficacious than asenapine and ziprasidone (see Table 1). On the CGI-S scores, all of the atypical antipsychotics were significantly more efficacious than placebo and clozapine was significantly more efficacious than asenapine and aripiprazole (see Table 1).

On weight gain, clozapine had the largest estimated weight gain relative to placebo, but was not significantly different from any other treatment due to large 95% credibility intervals precluding any meaningful conclusions (see Table 2). All of the atypical antipsychotics except for lurasidone, aripiprazole, and ziprasidone (and clozapine) had significantly greater weight gain than placebo. Olanzapine has significantly more weight gain than all other atypical antipsychotics (except clozapine). Lurasidone, aripiprazole, and ziprasidone had significantly less weight gain than asenapine, paliperidone, quetiapine, and risperidone. The weight gain profiles were varied across atypical antipsychotics, with lurasidone, aripiprazole, and ziprasidone having the least amount of weight gain.

Discontinuation rates were significantly lower for olanzapine, quetiapine, risperidone, and ziprasidone than placebo, aripiprazole, and paliperidone (see Table 2).

Lurasidone also had significantly lower discontinuation rates than aripiprazole and paliperidone.

There was limited information on EPS and akathisia for the different antipsychotics. Lurasidone, aripiprazole, risperidone, and ziprasidone had significantly greater rates of EPS than placebo and there were no significant differences between these atypical antipsychotics (see Table 3). Lurasidone had significantly greater rates of akathisia than placebo and there were no significant differences between the atypical antipsychotics on akathisia (see Table 3).

Table 1. Comparison of PANSS Total Score and CGI-S Score

(yellow)	PANSS Total Score Change
(blue)	CGI-S Total Score Change

**PLO**	**0.44 [0.22, 0.67]**	**0.35 [0.11, 0.59]**	**0.29 [0.06, 0.53]**	**1.60 [0.42, 2.78]**	**0.60 [0.02, 1.18]**	-	**0.41 [0.05, 0.77]**	-	**0.40 [0.12, 0.68]**
**-7.95 [-11.76, -4.16]**	**LUR**	-0.09 [-0.42, 0.24]	-0.15 [-0.48, 0.17]	1.16 [-0.04, 2.35]	0.16 [-0.47, 0.78]	-	-0.03 [-0.46, 0.39]	-	-0.05 [-0.40, 0.31]
**-7.21 [-10.78, -3.62]**	0.74 [-4.47, 5.97]	**ARI**	-0.06 [-0.39, 0.28]	**1.25 [0.05, 2.45]**	0.25 [-0.38, 0.88]	-	0.06 [-0.37, 0.50]	-	0.05 [-0.32, 0.42]
**-5.14 [-8.74, -1.54]**	2.81 [-2.42, 8.05]	2.06 [-3.02, 7.14]	**ASN**	**1.31 [0.11, 2.51]**	0.31 [-0.31, 0.93]	-	0.12 [-0.31, 0.55]	-	0.11 [-0.26, 0.47]
-	-	-	-	**CLO**	-1.00 [-2.03, 0.03]	-	-1.19 [-2.42, 0.05]	-	-1.20 [-2.41, 0.01]
**-16.01 [-26.45, -5.60]**	-8.06 [-19.17, 3.02]	-8.81 [-19.84, 2.22]	-10.86 [-21.93, 0.15]	-	**OLA**	-	-0.19 [-0.87, 0.50]	-	-0.20 [-0.84, 0.44]
**-5.59 [-9.90, -1.28]**	2.36 [-3.38, 8.09]	1.62 [-2.27, 5.50]	-0.45 [-6.04, 5.19]	-	10.41 [-0.85, 21.67]	**PAL**	-	-	-
**-8.94 [-15.58, -2.32]**	-0.98 [-8.64, 6.63]	-1.74 [-9.26, 5.76]	-3.80 [-11.35, 3.73]	-	7.06 [-5.29, 19.45]	-3.35 [-11.26, 4.53]	**QUE**	-	-0.01 [-0.47, 0.44]
**-12.31 [-17.65, -6.96]**	-4.36 [-10.90, 2.20]	-5.12 [-11.53, 1.33]	**-7.17 [-13.61, -0.73]**	-	3.69 [-7.31, 14.65]	-6.71 [-13.61, 0.13]	-3.37 [-11.88, 5.16]	**RIS**	-
-4.51 [-9.81, 0.77]	3.45 [-3.08, 9.95]	2.70 [-3.72, 9.09]	0.63 [-5.77, 7.03]	-	11.50 [-0.21, 23.22]	1.08 [-5.74, 7.89]	4.44 [-4.06, 12.90]	**7.80 [0.26, 15.32]**	**ZIP**

Results are reported as median differences in change scores with 95% credible intervals. Statistically significant results are bolded. In both the yellow portion (lower left) and blue portion (upper right) of the table, the column treatment is the reference treatment, meaning that the difference in change scores are relative to the column treatment. For example: in the yellow cell comparing risperidone to asenapine, asenapine is the reference treatment; the estimate indicates a -7.17 difference in change score for risperidone relative to asenapine (i.e. greater improvement in PANSS). Similarly, in the blue cell comparing aripiprazole to clozapine, clozapine is the reference treatment; the estimate indicates a 1.25 difference in change score for aripiprazole relative to clozapine (i.e. worse improvement in CGI-S). PLO=placebo. LUR=lurasidone. ARI=aripiprazole. ASN=asenapine. CLO=clozapine. OLA=olanzapine. PAL=paliperidone. QUE=quetiapine. RIS=risperidone. ZIP=ziprasidone.

Table 2. Comparison of Change in Body Weight (kg) and All-Cause Discontinuation

Change in Body Weight (kg)
All-Cause Discontinuation

**PLO**	1.88<br>[0.97, 3.63]	0.54<br>[0.24, 1.11]	1.09<br>[0.59, 1.96]	15.70<br>[0.43, 8232]	**2.97<br>[1.45, 6.16]**	0.47<br>[0.17, 1.23]	**2.20<br>[1.22, 3.94]**	**2.85<br>[1.45, 5.67]**	**1.71<br>[1.01, 2.88]**
0.28<br>[-0.26, 0.82]	**LUR**	**0.28<br>[0.10, 0.76]**	0.58<br>[0.24, 1.41]	8.43<br>[0.22, 4505]	1.58<br>[0.60, 4.22]	**0.25<br>[0.08, 0.81]**	1.17<br>[0.49, 2.82]	1.52<br>[0.59, 3.92]	0.91<br>[0.39, 2.11]
0.43<br>[-0.07, 0.92]	0.15<br>[-0.58, 0.88]	**ARI**	2.04<br>[0.79, 5.53]	29.85<br>[0.76, 16,090]	**5.56<br>[1.99, 16.40]**	0.89<br>[0.48, 1.64]	**4.11<br>[1.61, 11.08]**	**5.35<br>[1.97, 15.28]**	**3.19<br>[1.30, 8.32]**
**1.25<br>[0.75, 1.75]**	**0.98<br>[0.24, 1.71]**	**0.82<br>[0.12, 1.53]**	**ASN**	14.54<br>[0.38, 7709]	**2.72<br>[1.08, 7.06]**	0.43<br>[0.13, 1.35]	2.02<br>[0.88, 4.68]	**2.62<br>[1.07, 6.54]**	1.57<br>[0.71, 3.51]
4.08<br>[-0.10, 8.27]	3.81<br>[-0.42, 8.03]	3.65<br>[-0.56, 7.88]	2.83<br>[-1.39, 7.05]	**CLO**	0.19<br>[0.00, 6.29]	0.03<br>[0.00, 1.24]	0.14<br>[0.00, 5.24]	0.18<br>[0.00, 6.90]	0.11<br>[0.00, 4.10]
**3.90<br>[2.80, 4.99]**	**3.62<br>[2.41, 4.84]**	**3.47<br>[2.27, 4.67]**	**2.64<br>[1.44, 3.84]**	-0.19<br>[-4.23, 3.86]	**OLA**	**0.16<br>[0.05, 0.53]**	0.74<br>[0.30, 1.80]	0.96<br>[0.39, 2.34]	0.57<br>[0.23, 1.40]
**1.13<br>[0.66, 1.60]**	**0.85<br>[0.14, 1.57]**	**0.70<br>[0.19, 1.22]**	-0.12<br>[-0.80, 0.56]	-2.95<br>[-7.17, 1.26]	**-2.77<br>[-3.95, -1.58]**	**PAL**	**4.66<br>[1.51, 14.92]**	**6.04<br>[1.88, 20.43]**	**3.61<br>[1.21, 11.29]**
**2.41<br>[1.51, 3.32]**	**2.13<br>[1.08, 3.20]**	**1.99<br>[0.95, 3.02]**	**1.16<br>[0.12, 2.20]**	-1.67<br>[-5.96, 2.62]	**-1.48<br>[-2.90, -0.06]**	**1.28<br>[0.26, 2.31]**	**QUE**	1.30<br>[0.55, 3.08]	0.78<br>[0.35, 1.71]
**1.43<br>[0.60, 2.26]**	**1.16<br>[0.17, 2.14]**	**1.01<br>[0.04, 1.97]**	0.18<br>[-0.79, 1.14]	-2.66<br>[-6.89, 1.59]	**-2.47<br>[-3.72, -1.20]**	0.30<br>[-0.65, 1.25]	-0.98<br>[-2.21, 0.25]	**RIS**	0.60<br>[0.25, 1.41]
-0.10<br>[-0.78, 0.58]	-0.38<br>[-1.24, 0.49]	-0.53<br>[-1.37, 0.31]	**-1.35<br>[-2.20, -0.51]**	-4.19<br>[-8.43, 0.06]	**-4.00<br>[-5.28, -2.71]**	**-1.23<br>[-2.05, -0.41]**	**-2.52<br>[-3.65, -1.38]**	**-1.53<br>[-2.60, -0.47]**	**ZIP**

Results are reported as median differences in change scores for body weight (yellow portion, lower left) and as odds ratios for all-cause discontinuation (blue portion, upper right) with 95% credible intervals. Statistically significant results are bolded. In both the yellow portion (lower left) and blue portion (upper right) of the table, the column treatment is the reference treatment, meaning that the difference in change scores or odds ratios are relative to the column treatment. For example: in the yellow cell comparing olanzapine to lurasidone, lurasidone is the reference treatment; the estimate indicates a 3.62 kg difference in change score for olanzapine relative to lurasidone (i.e. olanzapine had 3.62 kg greater weight gain relative to lurasidone). In the blue cell comparing aripiprazole and olanzapine, olanzapine is the reference treatment; the estimate indicates that aripiprazole had 5.56 times greater odds of all-cause discontinuation compared to olanzapine. The results of olanzapine relative to aripiprazole can be obtained by inverting the odds ratio and CrI (i.e. 1/5.56 = 0.18). PLO=placebo. LUR=lurasidone. ARI=aripiprazole. ASN=asenapine. CLO=clozapine. OLA=olanzapine. PAL=paliperidone. QUE=quetiapine. RIS=risperidone. ZIP=ziprasidone.

Table 3. Comparison of Akathisia and Extrapyramidal Symptoms

Colour	Outcome
Yellow	Akathisia
Blue	Extrapyramidal Symptoms

**PLO**	**0.18 [0.04, 0.54]**	**0.24 [0.08, 0.59]**	0.45 [0.12, 1.29]	-	0.35 [0.10, 1.01]	**0.24 [0.03, 0.96]**	**0.06 [0.00, 0.37]**
**6.22 [1.67, 43.51]**	**LUR**	1.33 [0.27, 7.69]	2.52 [0.45, 15.41]	-	1.97 [0.36, 12.43]	1.33 [0.14, 10.26]	0.34 [0.01, 3.56]
2.34 [0.94, 7.06]	0.38 [0.04, 2.09]	**ARI**	1.89 [0.39, 8.81]	-	1.49 [0.31, 6.79]	1.00 [0.11, 5.94]	0.26 [0.01, 2.16]
7.90 [1.27, 209.10]	1.28 [0.09, 42.54]	3.41 [0.39, 99.66]	**ASN**	-	0.79 [0.15, 4.13]	0.53 [0.06, 3.54]	0.14 [0.00, 1.27]
4.34 [1.51, 15.37]	0.69 [0.08, 4.36]	1.85 [0.81, 4.45]		**PAL**	-	-	-
-	-	-	-	-	**QUE**	0.67 [0.07, 4.57]	0.17 [0.01, 1.62]
-	-	-	-	-	-	**RIS**	0.26 [0.01, 3.73]
2.28 [0.69, 10.73]	0.36 [0.04, 2.85]	0.98 [0.19, 5.79]	0.29 [0.01, 3.22]	0.53 [0.09, 3.42]	-	-	**ZIP**

Results are reported as odds ratios with 95% credible intervals. Statistically significant results are bolded. In both the yellow portion (lower left) and blue portion (upper right) of the table, the column treatment is the reference treatment, meaning that the difference in odds ratios are relative to the column treatment. For example: in the yellow cell comparing lurasidone to placebo, placebo is the reference treatment; the 6.22 indicates that lurasidone had 6.22 times greater odds of akathisia compared to placebo. The results of placebo relative to lurasidone can be obtained by inverting the odds ratio and CrI (i.e. 1/6.22=0.16). Similarly, in the blue cell comparing placebo to lurasidone, lurasidone is the reference treatment; the 0.18 indicates that placebo had 0.18 times greater odds of extrapyramidal symptoms compared to lurasidone. The results of placebo relative to lurasidone can be obtained by inverting the odds ratio and CrI (i.e. 1/0.18=5.56). PLO=placebo. LUR=lurasidone. ARI=aripiprazole. ASN=asenapine. CLO=clozapine. OLA=olanzapine. PAL=paliperidone. QUE=quetiapine. RIS=risperidone. ZIP=ziprasidone.
