# Supplementary material for: Lurasidone compared to other atypical antipsychotic monotherapies for adolescent schizophrenia: a systematic literature review and network meta-analysis
Source: Eur Child Adolesc Psychiatry. 2019 Nov 22;29(9):1195–205. doi: 10.1007/s00787-019-01425-2 (PMC7497364; doi:10.1007/s00787-019-01425-2)
Supplement: Supplementary file 5 — Supplementary material 5 (PDF 71 kb) [file 787_2019_1425_MOESM5_ESM.pdf]

Appendix 5. Random Effects Models

The random effects model results are included to document the differences from the fixed effects models reported in the main paper. The conclusions from the fixed effects and random effects models were consistent on change from baseline in PANSS total score, change from baseline in CGI-S score, extrapyramidal symptoms, akathisia, response, sedation, somnolence, change from baseline in total cholesterol, and change from baseline in triglycerides. While the estimates were similar, conclusions changed on some comparisons for change from baseline in weight (Table 3), all cause discontinuation (Table 4), discontinuation due to adverse events (Table 7), and serum glucose (Table 11). In each instance, comparisons between lurasidone and other antipsychotics went from being marginally significant to marginally non-significant. Results of the fixed effect models were reported as the primary results because they generally fit the data better based on the deviance information criterion (DIC), a deviance-based measure that penalizes models for complexity.

Table 1. Change from Baseline – PANSS Total Score

Comparison	Median Change	95% CrI Lower	95% CrI Upper
Aripiprazole vs. Placebo	-7.09	-11.01	-3.11
Asenapine vs. Placebo	-5.20	-9.23	-1.08
Lurasidone vs. Placebo	-7.94	-12.24	-3.74
Olanzapine vs. Placebo	-15.93	-26.08	-5.41
Paliperidone vs. Placebo	-5.55	-10.13	-0.91
Quetiapine vs. Placebo	-9.01	-15.97	-1.73
Risperidone vs. Placebo	-12.25	-17.95	-6.68
Ziprasidone vs. Placebo	-4.49	-10.21	1.14
Lurasidone vs. Aripiprazole	-0.84	-6.68	4.88
Lurasidone vs. Asenapine	-2.77	-8.68	3.06
Lurasidone vs. Olanzapine	7.97	-3.19	19.17
Lurasidone vs. Paliperidone	-2.38	-8.66	3.83
Lurasidone vs. Quetiapine	1.06	-7.32	9.21
Lurasidone vs. Risperidone	4.28	-2.71	11.41
Lurasidone vs. Ziprasidone	-3.45	-10.56	3.66

Note. No differences in conclusions from the fixed effects base case analysis.

Table 2. Change from Baseline – CGI-S Score

Comparison	Median Change	95% CrI Lower	95% CrI Upper
Aripiprazole vs. Placebo	-0.35	-0.74	0.04
Asenapine vs. Placebo	-0.29	-0.69	0.10
Clozapine vs. Placebo	-1.60	-2.90	-0.33
Lurasidone vs. Placebo	-0.44	-0.84	-0.06
Olanzapine vs. Placebo	-0.60	-1.30	0.10
Quetiapine vs. Placebo	-0.41	-0.89	0.07
Ziprasidone vs. Placebo	-0.40	-0.85	0.07
Lurasidone vs. Aripiprazole	-0.09	-0.65	0.46
Lurasidone vs. Asenapine	-0.15	-0.70	0.40
Lurasidone vs. Clozapine	1.16	-0.18	2.50
Lurasidone vs. Olanzapine	0.16	-0.63	0.93
Lurasidone vs. Quetiapine	-0.03	-0.64	0.57
Lurasidone vs. Ziprasidone	-0.04	-0.65	0.55

Note. No differences in conclusions from the fixed effects base case analysis.

Table 3. Change from Baseline – Weight

Comparison	Median Change	95% CrI Lower	95% CrI Upper
Aripiprazole vs. Placebo	0.48	-0.47	1.46
Asenapine vs. Placebo	1.21	0.32	2.17
Clozapine vs. Placebo	4.06	-0.42	8.54
Lurasidone vs. Placebo	0.28	-0.86	1.42
Olanzapine vs. Placebo	3.83	2.38	5.24
Paliperidone vs. Placebo	1.25	0.12	2.35
Quetiapine vs. Placebo	2.41	1.06	3.74
Risperidone vs. Placebo	1.50	0.33	2.75
Ziprasidone vs. Placebo	-0.10	-1.44	1.23
Lurasidone vs. Aripiprazole	-0.20	-1.70	1.27
**Lurasidone vs. Asenapine**	**-0.94**	**-2.43**	**0.50**
Lurasidone vs. Clozapine	-3.77	-8.41	0.83
Lurasidone vs. Olanzapine	-3.56	-5.33	-1.69
**Lurasidone vs. Paliperidone**	**-0.97**	**-2.56**	**0.62**
Lurasidone vs. Quetiapine	-2.12	-3.87	-0.36
**Lurasidone vs. Risperidone**	**-1.22**	**-2.92**	**0.41**
Lurasidone vs. Ziprasidone	0.38	-1.37	2.14

Note. Three differences in conclusions from the fixed effects base case analysis: lurasidone vs. asenapine, lurasidone vs. risperidone, and lurasidone vs. paliperidone went from being marginally significant to marginally insignificant.

Table 4. All Cause Discontinuation

**Comparison**	**Median Change**	**95% CrI Lower**	**95% CrI Upper**
Aripiprazole vs. Placebo	1.88	0.75	4.93
Asenapine vs. Placebo	0.91	0.41	2.08
Clozapine vs. Placebo	0.07	0.00	2.98
Lurasidone vs. Placebo	0.53	0.22	1.24
Olanzapine vs. Placebo	0.34	0.14	0.80
Paliperidone vs. Placebo	2.11	0.58	7.73
Quetiapine vs. Placebo	0.46	0.22	1.00
Risperidone vs. Placebo	0.35	0.15	0.77
Ziprasidone vs. Placebo	0.59	0.26	1.36
**Lurasidone vs. Aripiprazole**	**0.28**	**0.08**	**1.00**
Lurasidone vs. Asenapine	0.58	0.18	1.89
Lurasidone vs. Clozapine	7.99	0.16	2428.00
Lurasidone vs. Olanzapine	1.58	0.47	5.45
**Lurasidone vs. Paliperidone**	**0.25**	**0.05**	**1.18**
Lurasidone vs. Quetiapine	1.16	0.36	3.57
Lurasidone vs. Risperidone	1.55	0.47	5.16
Lurasidone vs. Ziprasidone	0.90	0.27	2.94

Note. Two differences in conclusions from the fixed effects base case analysis: lurasidone vs. aripiprazole and lurasidone vs. paliperidone went from being marginally significant to marginally insignificant.

Table 5. Extrapyramidal Symptoms

Comparison	Median Change	95% CrI Lower	95% CrI Upper
Aripiprazole vs. Placebo	4.25	1.00	19.24
Asenapine vs. Placebo	2.17	0.48	11.75
Lurasidone vs. Placebo	5.68	1.20	37.04
Quetiapine vs. Placebo	2.81	0.62	15.23
Risperidone vs. Placebo	4.26	0.71	38.93
Ziprasidone vs. Placebo	16.73	1.89	518.40
Lurasidone vs. Aripiprazole	1.33	0.16	14.31
Lurasidone vs. Asenapine	2.61	0.26	28.51
Lurasidone vs. Quetiapine	2.04	0.21	21.09
Lurasidone vs. Risperidone	1.34	0.09	17.31
Lurasidone vs. Ziprasidone	0.33	0.01	5.95

Note. No differences in conclusions from the fixed effects base case analysis.

Table 6. Akathisia

Comparison	Median Change	95% CrI Lower	95% CrI Upper
Aripiprazole vs. Placebo	2.44	0.51	13.85
Asenapine vs. Placebo	7.86	0.70	308.90
Lurasidone vs. Placebo	6.39	0.81	75.22
Paliperidone vs. Placebo	5.19	1.00	48.65
Ziprasidone vs. Placebo	2.31	0.27	23.93
Lurasidone vs. Aripiprazole	2.63	0.17	47.07
Lurasidone vs. Asenapine	0.80	0.01	26.25
Lurasidone vs. Paliperidone	1.23	0.06	22.64
Lurasidone vs. Ziprasidone	2.82	0.12	68.72

Note. No differences in conclusions from the fixed effects base case analysis.

Table 7. Discontinuation Due to Adverse Events

Comparison	Median Change	95% CrI Lower	95% CrI Upper
Aripiprazole vs. Placebo	2.48	0.56	14.91
Asenapine vs. Placebo	2.66	0.66	14.43
Lurasidone vs. Placebo	0.44	0.13	1.49
Olanzapine vs. Placebo	7.76	1.14	91.41
Paliperidone vs. Placebo	21.22	1.99	682.70
Quetiapine vs. Placebo	3.33	0.79	19.18
Risperidone vs. Placebo	2.40	0.51	15.06
Ziprasidone vs. Placebo	1.00	0.33	3.20
**Lurasidone vs. Aripiprazole**	**0.17**	**0.02**	**1.23**
**Lurasidone vs. Asenapine**	**0.16**	**0.02**	**1.05**
Lurasidone vs. Olanzapine	0.05	0.00	0.55
Lurasidone vs. Paliperidone	0.02	0.00	0.30
Lurasidone vs. Quetiapine	0.13	0.02	0.88
Lurasidone vs. Risperidone	0.18	0.02	1.30
Lurasidone vs. Ziprasidone	0.44	0.08	2.30

Note. Two differences in conclusions from the fixed effects base case analysis: lurasidone vs. asenapine and lurasidone vs. aripiprazole went from being marginally significant to marginally insignificant.

Table 8. Response

Comparison	Median Change	95% CrI Lower	95% CrI Upper
Asenapine vs. Placebo	1.75	0.79	3.91
Lurasidone vs. Placebo	2.34	1.04	5.23
Olanzapine vs. Placebo	4.69	1.14	20.42
Quetiapine vs. Placebo	1.28	0.57	2.77
Risperidone vs. Placebo	4.10	1.79	9.84
Lurasidone vs. Asenapine	1.33	0.43	4.17
Lurasidone vs. Olanzapine	0.50	0.09	2.51
Lurasidone vs. Quetiapine	1.82	0.61	5.78
Lurasidone vs. Risperidone	0.57	0.17	1.78

Note. No differences in conclusions from the fixed effects base case analysis.

Table 9. Sedation

Comparison	Median Change	95% CrI Lower	95% CrI Upper
Asenapine vs. Placebo	4.71	0.32	87.22
Lurasidone vs. Placebo	2.37	0.15	41.38
Olanzapine vs. Placebo	4.15	0.38	61.62
Quetiapine vs. Placebo	1.27	0.12	12.21
Risperidone vs. Placebo	2.60	0.07	106.60
Lurasidone vs. Asenapine	0.50	0.01	24.78
Lurasidone vs. Olanzapine	0.57	0.01	22.46
Lurasidone vs. Quetiapine	1.89	0.05	71.74
Lurasidone vs. Risperidone	0.94	0.01	87.05

Note. No differences in conclusions from the fixed effects base case analysis.

Table 10. Somnolence

Comparison	Median Change	95% CrI Lower	95% CrI Upper
Aripiprazole vs. Placebo	3.88	1.19	14.39
Asenapine vs. Placebo	3.26	0.87	13.01
Clozapine vs. Placebo	17.81	0.51	1435.00
Lurasidone vs. Placebo	2.09	0.53	9.22
Olanzapine vs. Placebo	15.00	1.75	556.80
Paliperidone vs. Placebo	4.86	1.31	23.36
Quetiapine vs. Placebo	5.98	1.50	27.44
Risperidone vs. Placebo	6.61	1.16	60.87
Ziprasidone vs. Placebo	3.58	0.79	17.14
Lurasidone vs. Aripiprazole	0.54	0.08	3.54
Lurasidone vs. Asenapine	0.64	0.09	4.64
Lurasidone vs. Clozapine	0.12	0.00	5.56
Lurasidone vs. Olanzapine	0.14	0.00	1.96
Lurasidone vs. Paliperidone	0.43	0.05	3.02
Lurasidone vs. Quetiapine	0.35	0.05	2.69
Lurasidone vs. Risperidone	0.31	0.02	3.10
Lurasidone vs. Ziprasidone	0.59	0.07	4.69

Note. No differences in conclusions from the fixed effects base case analysis.

Table 11. Change from Baseline – Serum Glucose

Comparison	Median Change	95% CrI Lower	95% CrI Upper
Aripiprazole vs. Placebo	3.53	-1.66	8.84
Asenapine vs. Placebo	3.56	-1.38	8.54
Lurasidone vs. Placebo	1.67	-3.50	6.88
Olanzapine vs. Placebo	4.51	-1.70	10.72
Paliperidone vs. Placebo	6.11	-1.57	14.00
Quetiapine vs. Placebo	0.85	-4.21	5.98
Ziprasidone vs. Placebo	-5.93	-13.23	1.37
Lurasidone vs. Aripiprazole	-1.86	-9.31	5.45
Lurasidone vs. Asenapine	-1.91	-9.05	5.28
Lurasidone vs. Olanzapine	-2.83	-10.92	5.28
Lurasidone vs. Paliperidone	-4.44	-13.95	4.87
Lurasidone vs. Quetiapine	0.83	-6.53	8.09
**Lurasidone vs. Ziprasidone**	**7.63**	**-1.35**	**16.56**

Note. One difference in conclusion from the fixed effects base case analysis: lurasidone vs. ziprasidone went from being statistically significant to statistically insignificant.

Table 12. Change from Baseline – Total Cholesterol

Comparison	Median Change	95% CrI Lower	95% CrI Upper
Aripiprazole vs. Placebo	4.26	-3.01	11.51
Asenapine vs. Placebo	9.10	1.73	16.44
Lurasidone vs. Placebo	8.94	1.27	16.90
Olanzapine vs. Placebo	8.54	-1.09	18.18
Paliperidone vs. Placebo	9.83	-0.76	20.45
Quetiapine vs. Placebo	15.60	7.30	24.05
Ziprasidone vs. Placebo	-2.98	-11.49	5.48
Lurasidone vs. Aripiprazole	4.72	-5.79	15.38
Lurasidone vs. Asenapine	-0.13	-10.79	10.64
Lurasidone vs. Olanzapine	0.38	-11.93	12.98
Lurasidone vs. Paliperidone	-0.90	-13.83	12.53
Lurasidone vs. Quetiapine	-6.71	-17.96	5.01
Lurasidone vs. Ziprasidone	11.97	0.46	23.65

Note. No differences in conclusions from the fixed effects base case analysis.

Table 13. Change from Baseline – Triglycerides

Comparison	Median Change	95% CrI Lower	95% CrI Upper
Aripiprazole vs. Placebo	3.61	-9.79	17.21
Asenapine vs. Placebo	8.21	-9.79	26.63
Lurasidone vs. Placebo	4.22	-10.19	18.90
Olanzapine vs. Placebo	37.01	12.27	61.74
Paliperidone vs. Placebo	11.54	-7.94	30.78
Quetiapine vs. Placebo	21.58	4.27	38.31
Ziprasidone vs. Placebo	8.82	-17.58	35.17
Lurasidone vs. Aripiprazole	0.46	-19.19	20.73
Lurasidone vs. Asenapine	-4.04	-27.10	18.93
Lurasidone vs. Olanzapine	-32.75	-61.30	-4.19
Lurasidone vs. Paliperidone	-7.32	-31.41	17.35
Lurasidone vs. Quetiapine	-17.32	-39.96	5.07
Lurasidone vs. Ziprasidone	-4.54	-35.25	25.65

Note. No differences in conclusions from the fixed effects base case analysis.
