# Supplementary material for: Lurasidone compared to other atypical antipsychotic monotherapies for adolescent schizophrenia: a systematic literature review and network meta-analysis
Source: Eur Child Adolesc Psychiatry. 2019 Nov 22;29(9):1195–205. doi: 10.1007/s00787-019-01425-2 (PMC7497364; doi:10.1007/s00787-019-01425-2)
Supplement: Supplementary file 6 — Supplementary material 6 (PDF 112 kb) [file 787_2019_1425_MOESM6_ESM.pdf]

Appendix 6. Sensitivity Analyses

Three sensitivity analyses were completed to assess the effects on the models of removing trials that had a lower average age, had a longer duration, or were inconsistent in direction of results. In some cases, a comparison on an outcome variable was dropped because it could not be examined without the excluded studies. For example, Shaw 2006 was the only study that included clozapine so comparisons between clozapine and lurasidone could not be made when Shaw 2006 was removed. The sensitivity analyses examined the main outcome variables: change from baseline in PANSS total score, change from baseline in CGI-S score, change from baseline in body weight, all-cause discontinuation, extrapyramidal symptoms, and akathisia. If the removed trials did not report the outcome variable, the sensitivity analysis was the same as the base case analysis and not reported.

In the first sensitivity analysis, the two trials with a lower mean age (Mozes 2006 and Shaw 2006) were removed. The main outcome variables reported in these two trials were change in PANSS total score (Mozes 2006 only), CGI-S score (Shaw 2006 only), weight change, and all-cause discontinuation. The only differences in statistical significance relative to the base case was that lurasidone no longer had statistically significantly less weight gain than risperidone after removing both trials with a lower mean age (see Tables 1 through 4).

In the second sensitivity analysis, the trials with longer duration were removed: first the 12-week trials and then both the 8- and 12-week trials. The

only 12-week trials were Mozes 2006 and Jensen 2008. These two trials reported results for PANSS total score (only Mozes 2006), weight change (only Mozes 2006), and all-cause discontinuation. The PANSS total score sensitivity analysis matched the earlier sensitivity analysis that excluded Mozes 2006. There were only two changes in conclusions after removing the 12-week trials: clozapine now had statistically significantly more weight gain than placebo and lurasidone no longer had statistically significantly less weight gain than risperidone (see Tables 1, 5 and 6).

In addition to the two 12-week trials, there were three 8-week trials: Shaw 2006, Findling 2015, and Savtiz 2015. Removing both the 8- and 12-week trials removed a total of 5 of the 13 trials from the network meta-analysis and affected all of the main outcome variables. There were two changes in conclusions relative to lurasidone: paliperidone and risperidone no longer had statistically significantly more weight gain than lurasidone (see Tables 7 through 12). There were two changes in conclusions relative to placebo: paliperidone no longer had statistically significantly greater change from baseline in PANSS total score and aripiprazole now had statistically significantly greater weight gain than placebo (see Tables 7 through 12).

In the third sensitivity analysis, the inconsistent Mozes 2006 and Jensen 2008 studies were individually removed. These two studies compared olanzapine and risperidone directly, but the results for all-cause discontinuation were descriptively in the opposite direction. Mozes 2006 reported results for PANSS total score (Table 1), weight change (Table 5), and all-cause discontinuation

(Table 14). The only changes in conclusions from the base case analysis after removing Mozes 2006 were on change in weight from baseline where clozapine now had statistically significantly greater weight gain than placebo and lurasidone no longer had statistically significantly less weight gain than risperidone. Jensen 2008 only reported results for all-cause discontinuation (Table 14) and removing Jensen 2008 did not alter any of the conclusions.

Across all of the sensitivity analyses, the results were largely similar with the base case analysis. The consistency of findings indicates the results were largely robust to the decision to include trials with younger mean age, longer duration, and inconsistent direction of findings for olanzapine versus risperidone.

Sensitivity Analysis 1: Removal of Two Trials with Lower Mean Age (Mozes 2006 and Shaw 2006)

Table 1. Change from Baseline in PANSS Total Score Excluding Trials with Lower Mean Age

Comparison	Median Change	95% CrI Lower	95% CrI Upper
Lurasidone vs. Placebo	-7.94	-11.76	-4.12
Aripiprazole vs. Placebo	-7.20	-10.77	-3.61
Olanzapine vs. Placebo	-12.47	-24.80	-0.16
Risperidone vs. Placebo	-13.02	-18.51	-7.54
Paliperidone vs. Placebo	-5.59	-9.89	-1.28
Quetiapine vs. Placebo	-8.96	-15.58	-2.32
Ziprasidone vs. Placebo	-4.51	-9.81	0.79
Asenapine vs. Placebo	-5.14	-8.74	-1.54
Lurasidone vs. Aripiprazole	-0.75	-5.98	4.49
Lurasidone vs. Olanzapine	4.53	-8.35	17.40
Lurasidone vs. Risperidone	5.07	-1.62	11.76
Lurasidone vs. Paliperidone	-2.36	-8.11	3.40
Lurasidone vs. Quetiapine	1.01	-6.64	8.70
Lurasidone vs. Ziprasidone	-3.43	-9.96	3.12
Lurasidone vs. Asenapine	-2.80	-8.04	2.45

Note. No differences in conclusions from the base case analysis. Only Mozes 2006 was removed because Shaw 2006 did not report change in PANSS total score.

Table 2. Change from Baseline in CGI-S Score Excluding Trials with Lower Mean Age

Comparison	Median Change	95% CrI Lower	95% CrI Upper
Lurasidone vs. Placebo	-0.45	-0.67	-0.22
Aripiprazole vs. Placebo	-0.35	-0.59	-0.11
Olanzapine vs. Placebo	-0.60	-1.18	-0.02
Quetiapine vs. Placebo	-0.41	-0.78	-0.05
Ziprasidone vs. Placebo	-0.40	-0.68	-0.12
Asenapine vs. Placebo	-0.29	-0.53	-0.06
Lurasidone vs. Aripiprazole	-0.09	-0.42	0.23
Lurasidone vs. Olanzapine	0.16	-0.46	0.77
Lurasidone vs. Quetiapine	-0.03	-0.46	0.39
Lurasidone vs. Ziprasidone	-0.05	-0.40	0.31
Lurasidone vs. Asenapine	-0.15	-0.48	0.17

Note. No differences in conclusions from the base case analysis. Only Shaw 2006 was removed because Mozes 2006 did not report change in CGI-S score.

Table 3. Change from Baseline in Weight Excluding Trials with Lower Mean Age

**Comparison**	**Median Change**	**95% CrI Lower**	**95% CrI Upper**
Lurasidone vs. Placebo	0.28	-0.26	0.81
Aripiprazole vs. Placebo	0.43	-0.07	0.92
Asenapine vs. Placebo	1.13	0.66	1.60
Olanzapine vs. Placebo	4.20	2.99	5.41
Risperidone vs. Placebo	1.28	0.41	2.15
Paliperidone vs. Placebo	1.25	0.75	1.75
Quetiapine vs. Placebo	2.41	1.50	3.32
Ziprasidone vs. Placebo	-0.10	-0.78	0.58
Lurasidone vs. Aripiprazole	-0.15	-0.88	0.58
Lurasidone vs. Asenapine	-0.85	-1.56	-0.14
Lurasidone vs. Olanzapine	-3.92	-5.24	-2.60
**Lurasidone vs. Risperidone**	**-1.00**	**-2.02**	**0.02**
Lurasidone vs. Paliperidone	-0.97	-1.71	-0.24
Lurasidone vs. Quetiapine	-2.13	-3.19	-1.08
Lurasidone vs. Ziprasidone	0.38	-0.49	1.24

Note. One change in conclusions from the base case analysis: lurasidone no longer has statistically significantly less weight gain than risperidone. Removed Mozes 2006 and Shaw 2006.

Table 4. All-Cause Discontinuation Excluding Trials with Lower Mean Age

Comparison	Odds Ratio	95% CrI Lower	95% CrI Upper
Lurasidone vs. Placebo	0.53	0.28	1.03
Asenapine vs. Placebo	0.92	0.51	1.69
Aripiprazole vs. Placebo	1.86	0.90	4.17
Paliperidone vs. Placebo	2.11	0.81	5.78
Olanzapine vs. Placebo	0.41	0.19	0.86
Quetiapine vs. Placebo	0.46	0.25	0.82
Ziprasidone vs. Placebo	0.59	0.35	0.99
Risperidone vs. Placebo	0.30	0.14	0.61
Lurasidone vs. Asenapine	0.58	0.24	1.41
Lurasidone vs. Aripiprazole	0.28	0.10	0.76
Lurasidone vs. Paliperidone	0.25	0.08	0.81
Lurasidone vs. Olanzapine	1.31	0.48	3.61
Lurasidone vs. Quetiapine	1.17	0.48	2.82
Lurasidone vs. Ziprasidone	0.91	0.39	2.10
Lurasidone vs. Risperidone	1.79	0.68	4.77

Note. No differences in conclusions from the base case analysis. Removed Mozes 2006 and Shaw 2006.

Sensitivity Analysis 2: Removal of Trials with Longer Duration

Sensitivity Analysis 2(a): Removal of 12-Week Trials (Mozes 2006 and Jensen 2008)

Table 5. Change from Baseline in Weight Excluding 12-Week Trials

**Comparison**	**Median Change**	**95% CrI Lower**	**95% CrI Upper**
Lurasidone vs. Placebo	0.28	-0.26	0.82
Aripiprazole vs. Placebo	0.43	-0.07	0.92
Asenapine vs. Placebo	1.13	0.66	1.60
**Clozapine vs. Placebo**	**4.38**	**0.18**	**8.62**
Olanzapine vs. Placebo	4.20	2.99	5.41
Risperidone vs. Placebo	1.28	0.41	2.14
Paliperidone vs. Placebo	1.25	0.75	1.75
Quetiapine vs. Placebo	2.41	1.50	3.32
Ziprasidone vs. Placebo	-0.10	-0.78	0.58
Lurasidone vs. Aripiprazole	-0.15	-0.88	0.58
Lurasidone vs. Asenapine	-0.85	-1.56	-0.14
Lurasidone vs. Clozapine	-4.10	-8.37	0.14
Lurasidone vs. Olanzapine	-3.92	-5.25	-2.59
**Lurasidone vs. Risperidone**	**-1.00**	**-2.02**	**0.03**
Lurasidone vs. Paliperidone	-0.97	-1.71	-0.24
Lurasidone vs. Quetiapine	-2.13	-3.19	-1.07
Lurasidone vs. Ziprasidone	0.38	-0.49	1.25

Note. Two changes in conclusions from the base case analysis: Clozapine now statistically significantly different from placebo and lurasidone no longer has statistically significantly less weight gain than risperidone. Only Mozes 2006 was removed because Jensen 2008 did not report change in weight.

Table 6. All-Cause Discontinuation Excluding 12-Week Studies

Comparison	Odds Ratio	95% Crl Lower	95% Crl Upper
Lurasidone vs. Placebo	0.53	0.28	1.04
Asenapine vs. Placebo	0.92	0.51	1.69
Aripiprazole vs. Placebo	1.86	0.90	4.20
Paliperidone vs. Placebo	2.11	0.81	5.79
Olanzapine vs. Placebo	0.35	0.15	0.80
Quetiapine vs. Placebo	0.43	0.23	0.79
Ziprasidone vs. Placebo	0.59	0.35	0.99
Risperidone vs. Placebo	0.38	0.17	0.82
Clozapine vs. Placebo	0.07	0.00	2.46
Lurasidone vs. Asenapine	0.58	0.24	1.41
Lurasidone vs. Aripiprazole	0.29	0.10	0.77
Lurasidone vs. Paliperidone	0.25	0.08	0.81
Lurasidone vs. Olanzapine	1.54	0.53	4.55
Lurasidone vs. Quetiapine	1.25	0.50	3.09
Lurasidone vs. Ziprasidone	0.91	0.39	2.11
Lurasidone vs. Risperidone	1.41	0.51	3.93
Lurasidone vs. Clozapine	8.25	0.20	4518.00

Note. No differences in conclusions from the base case analysis. Removed Mozes 2006 and Jensen 2008.

Sensitivity Analysis 2(b): Removal of 8- and 12-Week Trials (Mozes 2006, Jensen 2008, Shaw 2006, Findling 2015, and Savtiz 2015)

Table 7. Change from Baseline in PANSS Total Score Excluding 8- and 12-Week Studies

**Comparison**	**Median Change**	**95% Crl Lower**	**95% Crl Upper**
Lurasidone vs. Placebo	-7.95	-11.77	-4.11
Aripiprazole vs. Placebo	-7.05	-11.08	-3.02
Olanzapine vs. Placebo	-12.46	-24.78	-0.15
Risperidone vs. Placebo	-13.03	-18.50	-7.54
**Paliperidone vs. Placebo**	**-5.90**	**-11.96**	**0.15**
Quetiapine vs. Placebo	-8.92	-15.51	-2.28
Ziprasidone vs. Placebo	-4.50	-9.83	0.80
Lurasidone vs. Aripiprazole	-0.90	-6.46	4.64
Lurasidone vs. Olanzapine	4.50	-8.37	17.42
Lurasidone vs. Risperidone	5.07	-1.63	11.76
Lurasidone vs. Paliperidone	-2.05	-9.20	5.13
Lurasidone vs. Quetiapine	0.97	-6.67	8.60
Lurasidone vs. Ziprasidone	-3.44	-9.98	3.07

Note. One change in conclusions from the base case analysis: paliperidone no longer has statistically significantly greater reduction in PANSS total score than placebo. Removed Mozes 2006, Findling 2015, and Savitz 2015.

Table 8. Change from Baseline in CGI-S Score Excluding 8- and 12-Week Studies

**Comparison**	**Median Change**	**95% CrI Lower**	**95% CrI Upper**
Lurasidone vs. Placebo	-0.45	-0.67	-0.22
Aripiprazole vs. Placebo	-0.35	-0.59	-0.11
Olanzapine vs. Placebo	-0.60	-1.18	-0.02
Quetiapine vs. Placebo	-0.41	-0.77	-0.05
Ziprasidone vs. Placebo	-0.40	-0.68	-0.12
Lurasidone vs. Aripiprazole	-0.10	-0.43	0.23
Lurasidone vs. Olanzapine	0.15	-0.47	0.77
Lurasidone vs. Quetiapine	-0.03	-0.46	0.39
Lurasidone vs. Ziprasidone	-0.05	-0.40	0.31

Note. No differences in conclusions from the base case analysis. Removed Shaw 2006 and Findling 2015.

Table 9. Change from Baseline in Weight Excluding 8- and 12-Week Studies

Comparison	Median Change	95% CrI Lower	95% CrI Upper
Lurasidone vs. Placebo	0.28	-0.26	0.81
**Aripiprazole vs. Placebo**	**0.89**	**0.29**	**1.49**
Paliperidone vs. Placebo	0.74	0.20	1.29
Olanzapine vs. Placebo	4.20	2.99	5.41
Risperidone vs. Placebo	1.28	0.41	2.15
Quetiapine vs. Placebo	2.42	1.50	3.33
Ziprasidone vs. Placebo	-0.10	-0.78	0.59
Lurasidone vs. Aripiprazole	-0.61	-1.41	0.19
**Lurasidone vs. Paliperidone**	**-0.47**	**-1.24**	**0.30**
Lurasidone vs. Olanzapine	-3.92	-5.24	-2.60
**Lurasidone vs. Risperidone**	**-1.00**	**-2.02**	**0.02**
Lurasidone vs. Quetiapine	-2.14	-3.21	-1.07
Lurasidone vs. Ziprasidone	0.38	-0.49	1.24
Total residual deviance	25.99	18.28	38.80

Note. Three changes in conclusions from the base case analysis: aripiprazole had significantly more weight gain than placebo after removing 8- and 12-week study, while paliperidone and risperidone no longer have statistically significantly more weight gain than lurasidone. Removed Mozes 2006, Shaw 2006, Findling 2015, and Savitz 2015.

Table 10. All-Cause Discontinuation Excluding 8- and 12-Week Studies

Comparison	Odds Ratio	95% CrI Lower	95% CrI Upper
Lurasidone vs. Placebo	0.53	0.27	1.03
Aripiprazole vs. Placebo	1.86	0.90	4.18
Olanzapine vs. Placebo	0.35	0.15	0.80
Quetiapine vs. Placebo	0.43	0.23	0.80
Ziprasidone vs. Placebo	0.59	0.35	0.99
Risperidone vs. Placebo	0.38	0.17	0.82
Lurasidone vs. Aripiprazole	0.28	0.10	0.77
Lurasidone vs. Olanzapine	1.54	0.53	4.56
Lurasidone vs. Quetiapine	1.24	0.50	3.08
Lurasidone vs. Ziprasidone	0.91	0.39	2.11
Lurasidone vs. Risperidone	1.41	0.51	3.91

Note. No differences in conclusions from the base case analysis. Removed Mozes 2006, Jensen 2008, Shaw 2006, Findling 2015, and Savtiz 2015.

Table 11. Extrapyramidal Symptoms Excluding 8- and 12-Week Studies

Comparison	Odds Ratio	95% Crl Lower	95% Crl Upper
Lurasidone vs. Placebo	5.53	1.82	25.81
Aripiprazole vs. Placebo	4.23	1.72	12.96
Quetiapine vs. Placebo	2.83	0.98	10.72
Risperidone vs. Placebo	4.19	1.04	32.12
Ziprasidone vs. Placebo	15.66	2.81	390.40
Lurasidone vs. Aripiprazole	1.32	0.26	7.70
Lurasidone vs. Quetiapine	1.97	0.35	12.41
Lurasidone vs. Risperidone	1.33	0.14	10.47
Lurasidone vs. Ziprasidone	0.35	0.01	3.63

Note. No differences in conclusions from the base case analysis. Removed Findling 2015.

Table 12. Akathisia Excluding 8- and 12-Week Studies

Comparison	Odds Ratio	95% CrI Lower	95% CrI Upper
Lurasidone vs. Placebo	6.18	1.65	44.28
Aripiprazole vs. Placebo	1.82	0.68	6.02
Paliperidone vs. Placebo	25.71	2.00	11040.00
Ziprasidone vs. Placebo	2.28	0.68	10.92
Lurasidone vs. Aripiprazole	3.39	0.57	30.34
Lurasidone vs. Paliperidone	0.24	0.00	6.28
Lurasidone vs. Ziprasidone	2.72	0.35	26.95

Note. No differences in conclusions from the base case analysis. Removed Findling 2015 and Savitz 2015.

Sensitivity Analysis 3: Removal of Trials with Inconsistent Results (Mozes 2006 and Jensen 2008)

Sensitivity Analysis 3(a): Removal of Mozes 2006

Table 13. All-Cause Discontinuation Excluding Mozes 2006

Comparison	Odds Ratio	95% CrI Lower	95% CrI Upper
Lurasidone vs. Placebo	0.53	0.27	1.04
Asenapine vs. Placebo	0.92	0.51	1.69
Aripiprazole vs. Placebo	1.85	0.90	4.15
Paliperidone vs. Placebo	2.10	0.81	5.76
Olanzapine vs. Placebo	0.41	0.19	0.86
Quetiapine vs. Placebo	0.46	0.25	0.82
Ziprasidone vs. Placebo	0.59	0.35	0.99
Risperidone vs. Placebo	0.30	0.14	0.61
Clozapine vs. Placebo	0.08	0.00	2.82
Lurasidone vs. Asenapine	0.58	0.24	1.42
Lurasidone vs. Aripiprazole	0.29	0.10	0.77
Lurasidone vs. Paliperidone	0.25	0.08	0.81
Lurasidone vs. Olanzapine	1.32	0.48	3.63
Lurasidone vs. Quetiapine	1.17	0.48	2.84
Lurasidone vs. Ziprasidone	0.91	0.39	2.12
Lurasidone vs. Risperidone	1.79	0.68	4.79
Lurasidone vs. Clozapine	7.00	0.18	3916.00

Note. No differences in conclusions from the base case analysis.

Sensitivity Analysis 3(b): Removal of Jensen 2008

Table 14. All-Cause Discontinuation Excluding Jensen 2008

**Comparison**	**Odds Ratio**	**95% CrI Lower**	**95% CrI Upper**
Lurasidone vs. Placebo	0.53	0.27	1.03
Asenapine vs. Placebo	0.92	0.51	1.69
Aripiprazole vs. Placebo	1.87	0.90	4.20
Paliperidone vs. Placebo	2.12	0.81	5.85
Olanzapine vs. Placebo	0.29	0.13	0.63
Quetiapine vs. Placebo	0.43	0.23	0.80
Ziprasidone vs. Placebo	0.59	0.35	0.99
Risperidone vs. Placebo	0.44	0.21	0.92
Clozapine vs. Placebo	0.06	0.00	2.03
Lurasidone vs. Asenapine	0.58	0.24	1.41
Lurasidone vs. Aripiprazole	0.28	0.10	0.77
Lurasidone vs. Paliperidone	0.25	0.08	0.81
Lurasidone vs. Olanzapine	1.85	0.66	5.26
Lurasidone vs. Quetiapine	1.24	0.50	3.07
Lurasidone vs. Ziprasidone	0.91	0.39	2.10
Lurasidone vs. Risperidone	1.20	0.45	3.23
Lurasidone vs. Clozapine	9.74	0.25	5042.00

Note. No differences in conclusions from the base case analysis.
